# *Simira cordifolia* protects against metal induced-toxicity in *Caenorhabditis elegans*

**DOI:** 10.3389/fphar.2023.1235190

**Published:** 2023-11-15

**Authors:** Margareth Duran-Izquierdo, Lucellys Sierra-Marquez, Maria Taboada-Alquerque, Jesus Olivero-Verbel

**Affiliations:** Environmental and Computational Chemistry Group, School of Pharmaceutical Sciences, University of Cartagena, Zaragocilla Campus, Cartagena, Colombia

**Keywords:** biodiversity, natural products, mercury, lead, cadmium, alkaloids

## Abstract

*Simira cordifolia* (Hook.f.) Steyerm (Rubiaceae) is a vascular plant used in Northern Colombia as a source of pigments and wood. However, there is a lack of information regarding its pharmacology and toxicity. This research aimed to study the hydroalcoholic extract of *Simira cordifolia* as a protector against metal-induced toxicity in *Caenorhabditis elegans*. Preliminary phytochemical screening of the hydroalcoholic extract of *S. cordifolia* (HAE-Sc) was conducted using HPLC-ESI-QTOF. Wild-type N2 *C. elegans* larvae were exposed to different concentrations of HAE-Sc evaluating lethality (50–5000 μg/mL), growth, lifespan, resistance to heat stress, and its protective effect against Mercury (Hg)-, Lead (Pb)- and Cadmium (Cd)-induced lethality (50–1000 μg/mL). The main metabolites present in the extract were iridoids, β-carboline-alkaloids and polyphenols. Bioassays demonstrated that HAE-Sc exhibited low toxicity, with significant lethality (4.2% and 9.4%) occurring at 2500–5000 μg/mL. Growth inhibition reached up to 23.3%, while reproduction declined 13% and 17% at concentrations 500 and 1000 μg/mL, respectively. HAE-Sc enhanced the survival rate of the nematode under thermal stress by up to 79.8%, and extended the mean lifespan of worms by over 33% compared to control. The average lifespan was prolonged by 15.3% and 18.5% at 50 and 100 μg/mL HAE-Sc, respectively. The extract (1000 μg/mL) was able to reduce the death of *C. elegans* in the presence of heavy metals up to 65.9, 96.8% and 87% for Pb, Hg, and Cd, respectively. In summary, *S. cordifolia* shows potential protective effects in *C. elegans* against toxicity caused by heavy metals and heat.

## 1 Introduction

Metabolites from plants offer a diverse range of opportunities to obtain new lead compounds with a wide variety of beneficial biochemical properties. The future of this area is enormous, considering that less than 1% of higher plants have been studied for their pharmacological potential (Rehman et al., 2019; Inuki, 2020). In Colombia, there are about 15% of the plants with therapeutic uses registered for the world, some of them endemic, however, scientific research on them is scarce, despite the fact that several are in danger of extinction (Bernal and Mesa, 2022).

The biodiversity-based research has been focused on finding metabolites with the ability to alleviate the detrimental manifestations of diseases that currently have limited treatments or many adverse effects, such as neurodegenerative disorders, aiming to improve the quality of life for patients (Puri et al., 2022). Several neuronal-related pathologies display signs and symptoms that resemble those related to prenatal or postnatal exposure to environmental pollutants, in particular heavy metals (Nakamura and Lipton, 2009). In fact, some neurotoxic trace elements, such as Lead (Pb), Mercury (Hg), Cadmium (Cd) and Arsenic (As), have been associated to Parkinson’s and Alzheimer’s diseases, (Nabi and Tabassum, 2022). These non-essential toxic elements exert their effects at low concentrations through multiple mechanisms of toxicity, among which oxidative damage stands out, since they generate high levels of reactive oxygen species (ROS) (Kim et al., 2019; Song et al., 2019).

In Colombia, heavy metal contamination in water, soil, and foods is an issue that affects the health of rural communities, (farmers and indigenous people). However, access to therapies against signs and symptoms in rural communities is precarious and the use of natural products derived from plants with antioxidant and antineurotoxic properties is a plausible alternative for managing the signs and symptoms of these diseases (Amadi et al., 2019). Among the huge arsenal of medicinal plants from Colombian biodiversity, *S. cordifolia* (Hook.f.) Steyerm [Rubiaceae] (*Simira cordifolia*)*, is* a vascular plant (Rubiaceae) popularly known as “Pijiño,” “Paraguatán,” “Brasil de Loma,” and “Brasilete Colorado.” It has been reported for dry forest ecosystems, growing in primary forests and also in intervened areas, from the sea level up to 1000 m.a.s.l. (Mendoza et al., 2004). *Simira cordifolia* leaves have also been commonly used as a source of pigments, usually isolated employing bark decoction methods, (dry or fresh) (Polesna et al., 2011). Despite its number of uses, the chemical composition and phytochemical properties has been poorly documented. In the process of generating knowledge about the bioactivities of natural products, *in vivo* assays are key to provide the basis for efficacy, adverse effects and modes of action that are relevant to humans, (Ha et al., 2022). They also offer insights on the relationship between chemical components and the biochemical pathways involved in diseases (Pohl and Kong Thoo Lin, 2018).

There are various *in vitro* and *in vivo* biological models available for the evaluation of the bioactivity, beneficial properties, screen for pharmacologically active compounds and toxicity of phytochemicals of foods and plants and to study their mechanisms of action (Ayuda-Duran et al., 2020). Among these, the nematode *Caenorhabditis elegans* has significant advantages (Xiong et al., 2017; Ha et al., 2022). *Caenorhabditis elegans* shares homology with mammalian systems and has approximately 40% of genes related to human diseases (Martins et al., 2022). It possesses complete systems, muscles, basic physiological processes, and oxidative stress responses comparable to those of higher organisms (Zhu et al., 2022), as well as high sensitivity to low concentrations of environmental toxic agents (Stylianou et al., 2019). It offers various genetic tools, a multitude of transgenic strains for diverse genes of interest, and a fully mapped neural system. Other features such as its lifecycle, morphology, reproduction rate, and simplicity are noteworthy for assessing toxic or beneficial effects in this animal model. *Caenorhabditis elegans* has demonstrated good predictive capacity when compared to mammalian models (Markaki and Tavernarakis, 2020), which has allowed it to be a potential model for assessing the toxicity of various products in their early stages of development, serving as a bridge between *in vitro* assays and mammalian tests (Hunt et al., 2020).

Numerous studies have reported the adverse effects of nematode exposure to heavy metals. The biotoxicities on *C. elegans* of Hg and Pb are strong, as they induce damaging effects in *C. elegans*, impacting antioxidative enzymes, lifespan, and reproductive toxicity (Huang et al., 2015). For instance, Lead (Pb) exposure has been linked to early neurotoxic sequelae, methylmercury (MeHg) causes alterations in body length, locomotion, and neurotoxicity, and Cadmium (Cd) leads to reproductive impairments and oxidative stress, ultimately resulting in Cd-induced neurotoxicity (Agarrayua et al., 2023).

The aim of this research was to study the hydroalcoholic extract of *S. cordifolia,* its constituents and biological effects as a protector against metal-induced toxicity using the *C. elegans* model, which is widely used in this context.

## 2 Materials and methods

### 2.1 Chemicals and reagents

All reagents used were of analytical grade. Acetonitrile (ACN) and water (all of LC/MS grade) were purchased from Merck (Darmstadt, Germany); formic acid (98%), peptone, KH_2_PO_4_, and agar bacteriology grade were obtained from Panreac AppliChem (Darmstadt, Germany); DMSO and Heavy metals were purchased from Sigma-Aldrich (St. Louis, MO, United States).

### 2.2 Plant material

The bark of *S. cordifolia* were collected from the forest of “Los Montes de Maria”, Colombian Caribbean during the dry season, and identified at the Herbarium University of Cordoba, Colombia. A voucher specimen (No. HUC-8451) was deposited in the Herbarium University of Cordoba. Plant collection permit was obtained from the Cardique (Cartagena, Colombia) under Resolution 0551 dated 14 June 2014. The use of *S. cordifolia* by communities is basically restricted to the bark, as it is the main source of the pigments.

### 2.3 Preparation of hidroalcoholic extract

The plant material was processed in the laboratories at the University of Cartagena. The bark was quickly washed with Milli-Q water and dried at room temperature. The dried plant material was ground, and dried under vacuum. Next 160 g (dried plant material) was subjected to extraction (maceration) for 48 h with a mixture containing ethanol (99.9%) and Milli-Q water in a 70:30 ratio (Vol/Vol) in 1.2 L mixure. After 24 h, the extract was filtered with Whatman paper, and the plant material was again subjected to the hydroalcoholic extraction for another 24 h. The combined extracts were rotary evaporated up to 40 mL and then freeze dried, (Duran-Izquierdo, et al., 2022). The hydro-alcoholic extract of the plant (HAE-Sc) was lyophilized obtaining a final yield of 5.6%. Finally, the extract was prepared as a stock solution of 100 mg/mL in DMSO, filtered through a 0.20 μm pore size syringe filter and stored at −20°C until further use.

### 2.4 Phytochemical profiling using high-performance liquid chromatography (HPLC-QTOF-MS/MS) conditions

The freeze-dried extract of *S. cordifolia* (6 mg) was diluted in 3 mL of a mixture of H_2_O and CH_3_CN 50:50. Diluted extract was vortexed and centrifuged at 16.000 ×g for 8 min, the supernatant was filtered (0.20 μm particle size) and transferred to autosampler vials. High-performance liquid chromatography/quadrupole time-of-flight mass spectrometry (HPLC-QTOF-MS/MS) was used to separate and characterize the metabolites. Chromatographic separation was achieved using a 1,260 Infinity HPLC (Agilent Technologies), equipped with a Variable Wavelength Detector (VWD), employing a InfinityLab Poroshell 120 EC-C18 column, of 4.6 × 100 mm, x 2.7 μm particle size (Agilent technologies, United States). The mobile phase flow rate was 0.3 mL/min, consisting of H_2_O (A) and CH_3_CN (B), with 0.1% HCOOH. Analysis started with 95:5 A: B, held for 1 min, followed by changed linearly up to 5:95 in 9 min, with a hold to 4 min; Then, changed to 100% acetonitrile in 1 min and it was stable for 3 min. Column re-equilibration was performed by returning to 95:5 A: B at minute 23 and holding until 26 min. The mass analysis was obtained using a Quadrupole-Time of Flight tandem mass spectrometer 6530 Q-ToF detector (Agilent Technologies), with Electrospray Ionization (ESI), operated in positive ion mode. The conditions for the mass detector were as follows: Capillary voltage +3.5 kV, nitrogen gas temperature 320°C, drying gas flow rate 8.0 L/min, nebulizer gas pressure 35 psig, fragmentor voltage 135 V, skimmer 65 V, and OCT RF 750 V. MS/MS Data Acquisition mode was used to assist compound identification. Mass range in MS and MS/MS experiments were set at m/z 100–1,200 and 50–1,200 at 3 spectra/s, respectively. MS and MS/MS data were collected using Agilent MassHunter Acquisition software (version 10.1). The data obtained was processed with the Agilent MassHunter Qualitative Analysis 10.0.

### 2.5 *Caenorhabditis elegans* strains, handling conditions and synchronization

*Caenorhabditis elegans (C. elegans)* wild-type N2 and *E. coli* strains (*Escherichia coli* OP50) were obtained from the Caenorhabditis Genetics Centre (CGC) of the University of Minnesota (Minneapolis, MN, United States). The worms were maintained at 20°C on the nematode growth medium (NGM), seeded with *E. coli* OP50. Strain was cultivated under standard laboratory conditions following standard protocols (www.wormbook.org). To obtain nematode in the same growth period, age synchronization was done by the egg-prep method from the CGC, employing a bleaching solution (0.45 M NaOH, 5% HOCl). Age-synchronized worms were cultured on NGM plates containing *E. coli* OP50 as a food source, and kept in a 20°C incubator until use. OP50 was grown in Luria-Bertani (LB) broth for 24 h in an incubator at 37°C (Xu et al., 2023).

### 2.6 Biological assays

To determine the biological activity of the HAE-*Sc* extract on *C. elegans*, we first evaluated the physiological changes of the worm after exposure to the extract, checking for lethality, growth, reproduction, motility, lifespan and response to heat stress were assessed. Subsequently the toxic effect of different concentrations of Mercury (Hg), Lead (Pb) and Cadmium (Cd) were evaluated, and then assessing the protective effect of HAE-Sc extract against Hg-, Pb- and Cd-induced lethality in the worm. The plant extract was dissolved in dimethyl sulfoxide (DMSO) at a stock concentration of 5000, 2500, 1000, 500, 250, 100, and 50 mg/mL, and then added to the NGM agar or K-medium at a final concentration of 5000, 2500, 1000, 500, 250, 100, and 50 μg/mL, respectively.

#### 2.6.1 Lethality

Synchronized L4 worms were exposed in 96-well plates (12 ± 2 worms/well) for 24 h at 20°C. The control group obtained 1% (v/v) DMSO, 50, 100, 250, 500, 1000, 2500 and 5000 μg/mL HAE-Sc dissolved in DMSO. The number of living and dead worms was counted. The nematodes were considered dead when they failed to respond to touch using a platinum loop (Nagar et al., 2020).

For the endpoint tests of growth, locomotion, and reproduction, the concentrations that exhibited low lethality in *C. elegans* (50–1000 μg/mL) were evaluated. Regarding the analysis of lifespan and resistance to thermal stress, concentrations of 50–500 μg/mL were assessed.

#### 2.6.2 Total brood size assays

The synchronized N2 worms were treated either the vehicle (0.1% DMSO; control) or HAE-Sc at different concentrations and were transferred to new NGM Petri dishes (a single worm per NGM plate) seeded with *E. coli* OP50 and transferred every 24 h until the 72 h, just before the egg-laying period. The egg-bearing plates were examined following incubation at 20°C for 72 h, and the total number of eggs or larvae was counted as the progeny size of the nematode (Jin et al., 2019).

#### 2.6.3 Body length assays

After exposure to control (1% DMSO) or HAE-Sc test solutions for 48 h at 20°C, N2 nematodes (L1 stage) were immobilized by heating them at 50°C for 15 min. The length of randomly selected worms (*n* = 30) was then measured for each concentration using a Nikon stereomicroscope and ImageJ software (NIH, Bethesda, MD, United States) (Miao et al., 2020).

#### 2.6.4 Locomotion measurement

Thirty L4 nematodes were exposed to control (1% DMSO) or different HAE-Sc concentrations on NGM without *E. coli* OP50 for 24 h at 20°C. After exposure, the number of body bends was counted following the recommendations published by Sawin et al., 2000.

#### 2.6.5 Lifespan analysis

The worms synchronized to the L4 stage were dispensed into 96-well plates with 12 worms per plate on NGM, which contained inactivated OP50 (60°C for 35 min). The plates were supplemented with 5-fluoro-2′-deoxyuridine (FUdR) to inhibit oviposition and hatching, preventing self-fertilization. Subsequently, the worms were treated with 1% DMSO (control) or different concentrations HAE-Sc. The worms were scored every 24 h until all of them were dead, indicated by their lack of response to radial and physical stimuli. Any worms that disappeared from the plate or died prematurely due to internal hatching or vulval rapture were excluded from the analysis (Zhang et al., 2019).

#### 2.6.6 Stress resistance assay (heat shock)

Synchronized L1 wild-type worms were cultured for 48 h at 20°C in 24-well plates with either 1% DMSO or HAE-Sc (50–500 μg/mL). Subsequently, the worms were exposed to 37°C for 2 h and then shifted back to 20°C. The number of viable worms was counted every 12 h until all the worms in the control group died. Each experiment used a minimum of 90 worms (Jin et al., 2019).

#### 2.6.7 Survival under metals-induced toxicity

The experimental design and test procedures were conducted following the previously described methods with minor modifications (Duran-Izquierdo et al., 2022). Synchronized L4 larvae stage of wild-type N2 worms were exposed to vehicle control or increasing concentrations, of heavy metals, namely, Hg, Pb and Cd. Another group of worms was exposed to co-treatments containing 1000 μg/mL of the extract, with the same metal concentration. The exposure took place for 24 h at 20°C in 96-well tissue culture plates (Corning Incorporated, United States). After the 24-h exposure period, the worms were observed under a dissection microscope and classified as either alive, if they were moving, or dead, if unresponsive to gentle probing. The Lethality data were used to estimate LC_50_ values.

All experiments described here were performed three times with at least three replicates for each treatment.

### 2.7 Data analysis

All data were expressed as mean ± standard error of the mean (SEM). Normality and equality of variances were assessed using the Shapiro-Wilk and Barlett test, respectively. Differences between groups were determined using analysis of variance (one-way ANOVA) followed by Dunnett’s test for comparisons against control. If normality was not met, Kruskal Wallis followed by Dunn’s Test were used instead. Values of *p* ≤ 0.05 were considered statistically significant. LC_50_ for metal mixtures were calculated using Probit analysis. The data of the lifespan assays were processed using a Kaplan-Meier analysis and log-rank test. Statistical analyses were performed using GraphPad Prism 5™ Software (GraphPad Software, Inc., San Diego, CA, United States).

## 3 Results

### 3.1 Chemical composition of hydroalcoholic extract of *Simira cordifolia* (HAE-Sc)

**FIGURE 1:**
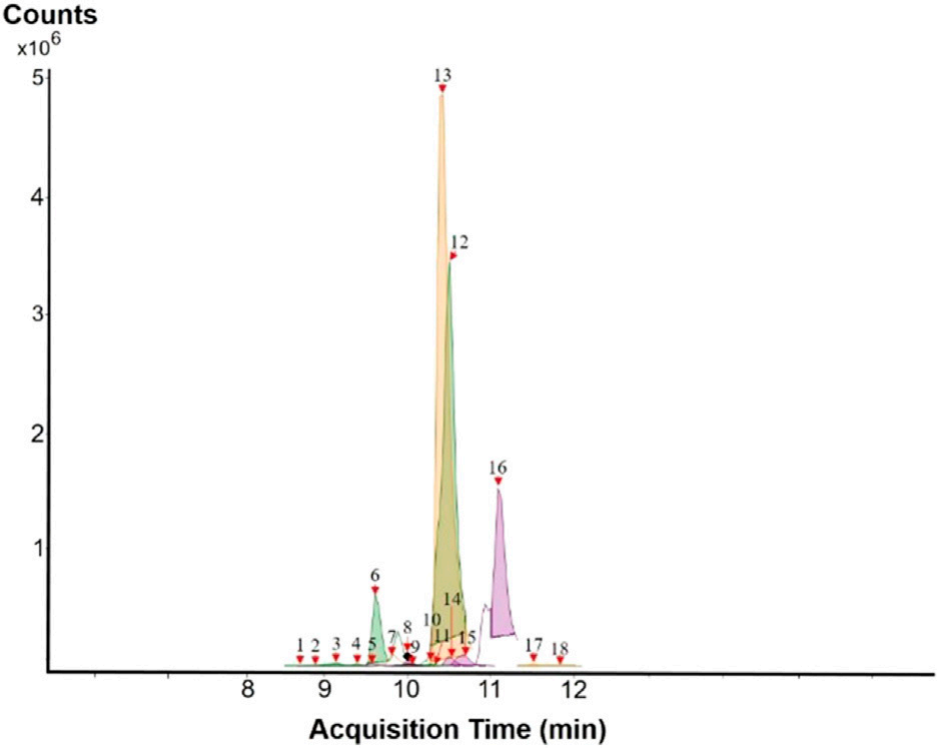
Extracted ion current (EIC) chromatogram of HAE-Sc.

The results of the HPLC-QTOF-MS/MS analysis for the hydro-alcoholic extract of *S. cordifolia* are shown in Figure 1 and Table 1. The monoisotopic mass characteristics for positive ions of the compounds tentatively identified, and fragment spectrum results (MS/MS) are presented in Supplementary Table S1 and Supplementary Figure S1). The tentative annotations belong to various compounds classes, including iridoids (diderroside, loganin, sweroside, nepetaside, 6-O-methoxyl cinnamoyl scandoside), isoquinoline alkaloid (anhalonidine, alamarine), polyphenol (chlorogenic acid, 5-caffeoylquinic acid, 3-feruloylquinic acid), *β*-carboline alkaloid (harmol, lyalosidic acid, capitelline, harman) and indole alkaloid (strictosidine, 19-epi-ajmalicine, Strictosamide). An ion represented by peak 17, could not be distinguished by its masses and fragmentation profiles; however, they showed fragment ions or losses typical of indole alkaloid class.

**TABLE 1:** Exact mass characteristic positive ions of compounds identified by HPLC-ESI-QTOF in *S. Cordifolia* extract.

No.	$t_{R}$ , min	Tentative annotation	Structure	Formula	Observed ion	Experimental mass	Calculated mass	∆ ppm
1	8.754	Diderroside	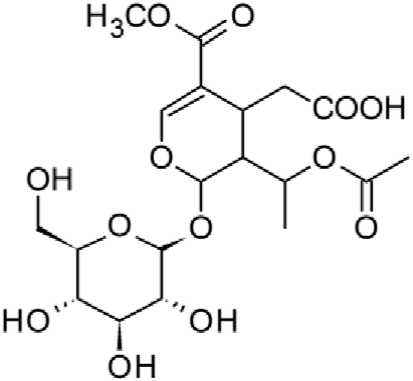	C_19_H_28_O_13_	[M + Na]+	464.1520	464.1530	−2.15
2	8.931	Loganin	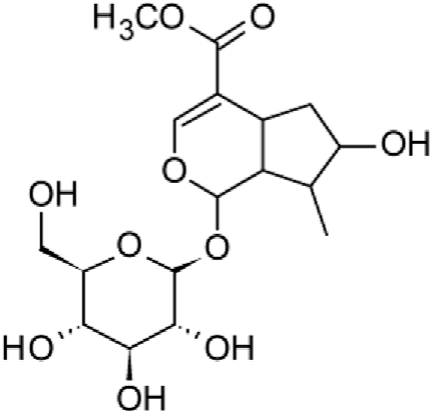	C_17_H_26_O_10_	[M + Na]+	390.1516	390.1526	2.56
3	9.054	Anhalonidine	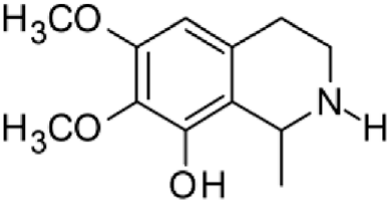	C_12_H_17_NO_3_	[M + H]+	223.1208	223.1208	0.0
4	9.41	Chlorogenic acid	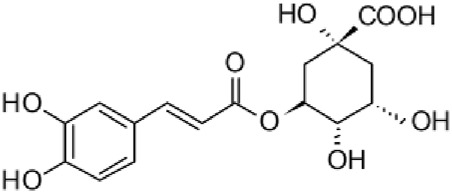	C_16_H_18_O_9_	[M + H]+	354.0949	354.0951	−0.56
5	9.602	Harmol	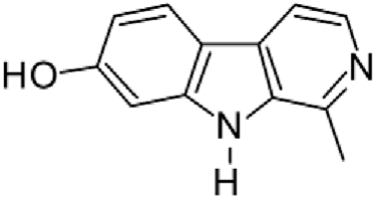	C_12_H_10_N_2_O	[M + H]+	198.0790	198.0793	−1.51
6	9.671	Lyalosidic acid	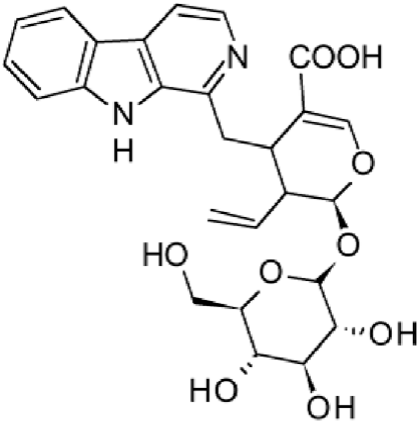	C_26_H_28_N_2_O_9_	[M + H]+	512.1808	512.1795	2.54
7	9.795	Chlorogenic acid isomer	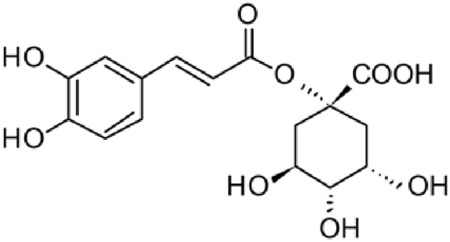	C_16_H_18_O_9_	[M + H]+	354.0949	354.0951	−0.56
8	10.01	3-Feruloylquinic acid	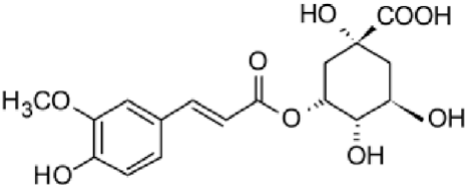	C_17_H_20_O_9_	[M + H]+	368.1117	368.1107	2.72
9	10.027	Sweroside	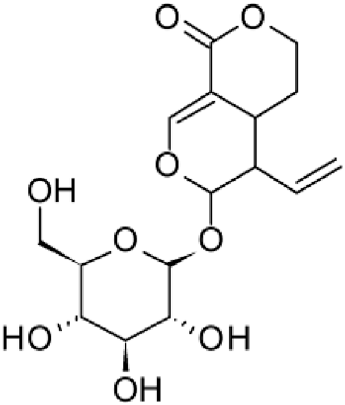	C_16_H_22_O_9_	[M + H]+	358.1269	358.1264	1,40
10	10.281	Nepetaside	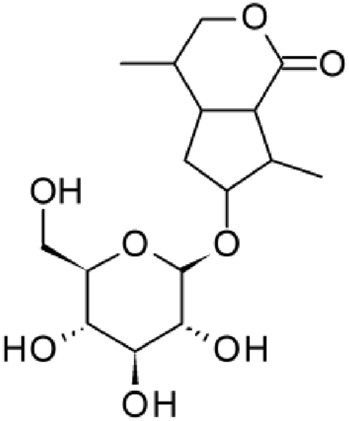	C_16_H_26_O_8_	[M + H]+	346.1623	346.1628	−1.44
11	10.32	6-O-Methoxyl cinnamoyl scandoside	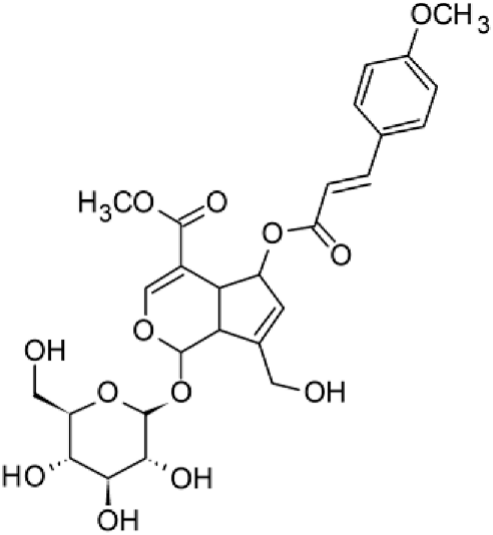	C_27_H_32_O_13_	[M + H]+	564.1864	564.1843	3.72
12	10.354	β -carboline alkaloid type	No ID	C_19_H_20_N_2_O_3_	[M + H]+	324.1837	324.1838	−0.31
13	10.431	Harman	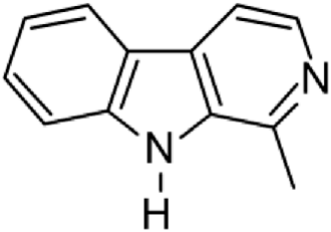	C_12_H_10_N_2_	[M + H]+	182.0847	182.0844	1.64
14	10.511	Indole glucoalkaloid type	No ID	C_27_H_34_N_2_O_9_	[M + H]+	530.2267	530.2264	0.56
15	10.712	Alamarine	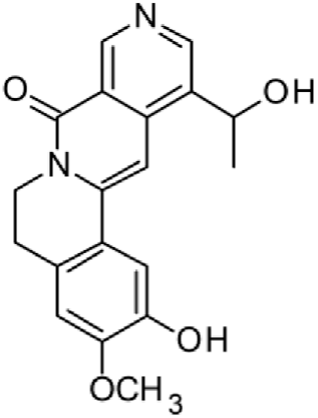	C_19_H_18_N_2_O_4_	[M + H]+	338.1271	338.1267	1.18
16	10.971	19-Epi-Ajmalicine	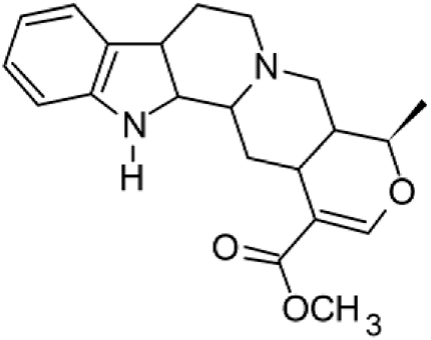	C_21_H_24_N_2_O_3_	[M + H]+	352.1795	352.1787	2.27
17	11.552	No ID	No ID	C_19_H_24_N_2_O	[M + H]+	296.1888	296.1889	−0.33
18	11.84	Strictosamide	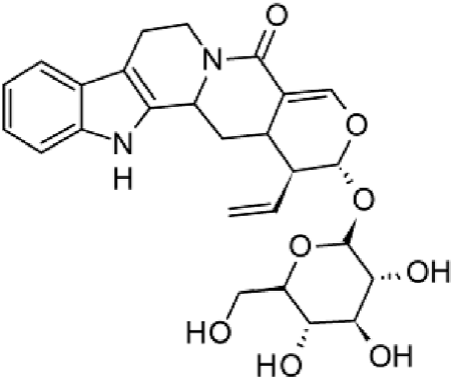	C_26_H_30_N_2_O_8_	[M + H]+	498.2019	498.2002	3.41

Peaks 1, 2, 9, 10 and 11 correspond to iridoids; peak 9 was tentatively identified as sweroside by comparison of several fragment ions (m/z: 197.0801, 179.0700 and 127.0382) representative of the tandem mass spectra reported for the same molecule in mass bank. Peaks 4, 7 and 8 had molecular loss of C_7_H _12_O_6_, which corresponds to quinic acid, linked by an ester to caffeic acid to form chlorogenic acids, such as chlorogenic acid, and 3-feruloylquinic acid. These annotations were confirmed by comparison with the mass spectra reported in the Mass Bank Database (MBD) for these molecules (Mass Bank Code [MBC] for chlorogenic acid and 3-feruloylquinic are FIO00622 and CCMSLIB00000853669, respectively.

Peak 13 was tentatively annotated as harman, a *β*-carboline alkaloid reported in Rubiaceae species, with similarities between its mass spectrum and spectral information reported in MBD, with MBC FIO00111 (Martins and Nunez, 2015). Interestingly, the monoisotopic mass of peak 13 (182.0847), was detected as a fragment ion in the mass spectra of peaks 12 and 16, suggesting that they have the basic Harman skeleton of C_12_H_10_N_2_, characteristic of *β*-carboline alkaloids (Phuong et al., 1998). Peak 5 had similar fragmentation characteristics to peak 13, indicating this molecule may also share the beta-carboline biosynthetic pathway that produces the tryptophan-derived *β*-carboline alkaloids. Peak 6 had fragmentation characteristics (m/z: 307.1444) similar to peak 16, showing this molecule also belongs to *β*-carboline alkaloids, but with the loss of a hexoside glycoside, C_6_H_10_O_5_, leading to believe that peak 6 is the *β*-carboline glucoalkaloid lyalosidic acid, also reported in rubiaceaes species (Martins and Nunez, 2015). Strictosamide, was another glycosylated beta-carboline detected in the extract of *S. cordifolia* with peak 18. This peak showed similar ion fragments (m/z: 499.1859; 337.1530; and 171.0911), as the mass spectrum of strictosamide reported by MBD as MDC CCMSLIB00004680054.

Peaks 4, 7 and 8 were annotated as polyphenols due to their fragmentation characteristics, quite similar to molecules reported in the literature as chlorogenic acid (MBC: FIO00622) and its isomer 3-feruloylquinic acid (MBC: CCMSLIB00000853669), respectively.

### 3.2 Lethality of HAE-Sc on *C. elegans*

**FIGURE 2:**
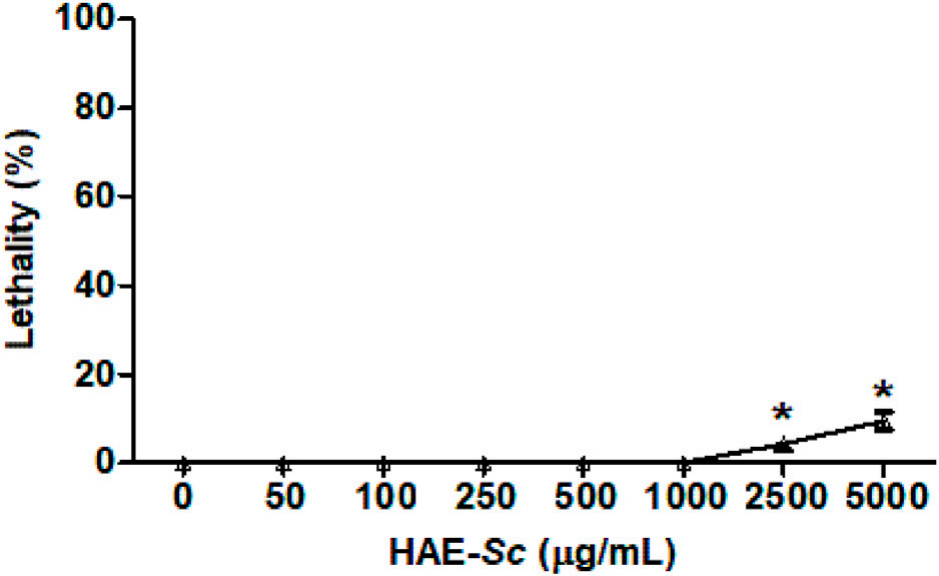
Lethality activity of HAE-Sc on *C. elegans* after 24 h exposure. *Significant difference (*p* < 0.05) when compared to control (0 μg/mL).

Lethality of HAE-Sc on *C. elegans* is presented in Figure 2. At the highest tested concentrations, 2500 and 5000 μg/mL, HAE-Sc exhibited 4.2% and 9.4% lethality, respectively. Concentrations lower than 1000 μg/mL did not display any lethality. Following these results, the concentration of 1000 μg/mL was selected to perform protection against heavy metal-induced toxic effects.

### 3.3 Physiological endpoints (reproduction, body length and motility)

**FIGURE 3:**
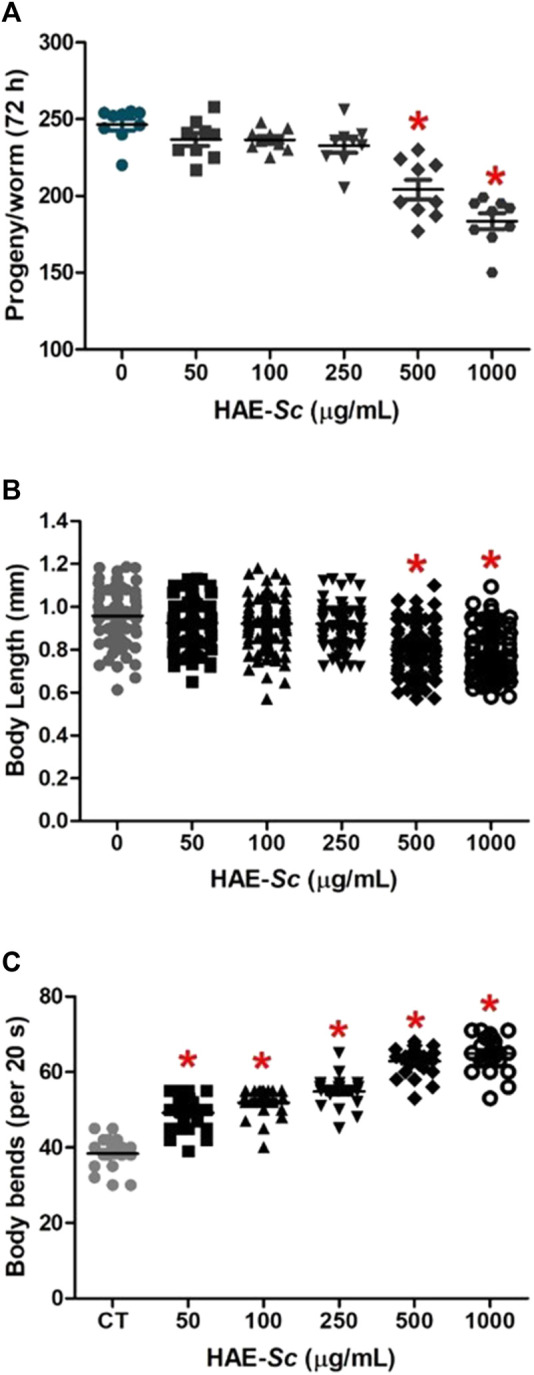
Effects of HAE-SC on physiological endpoints in *C. elegans*. Reproduction **(A)**, body length **(B)**, and body bends **(C)**. *Significant difference when compared to control (*p* < 0.05). CT = Control group (0 μg/mL).

The effects of HAE-Sc on physiological endpoints in *C. elegans* are shown in Figure 3. For total brood size (progeny per worm), after 24 h of exposure, HAE-Sc induced reduction of egg deposition only at 500 and 1000 μg/mL treatments, with 13% and 17% decrease, respectively compared to the control (Figure 3A). The body length of worms exposed to HAE-Sc are shown in Figure 3B. Statistical differences in mean values were observed between the control group (0.1% DMSO) and those treated with 500 and 1000 μg/mL HAE-Sc (Figure 3B), with reductions of 18.7% and 23.3%, respectively. Worms exposed to HAE-Sc exhibited a significant increase in the number of body bends (“locomotion rate”) (Figure 3C). The average number of body bends per worm in the control group was 38.3 ± 1.0/20 s. Compared to this group, the worms exposed to HAE-Sc (50–1000 μg/mL) experienced significant mean increases in body bends, from 21.9 (50 μg/mL) to 40.8% (1000 μg/mL).

### 3.4 Lifespan analysis

**FIGURE 4:**
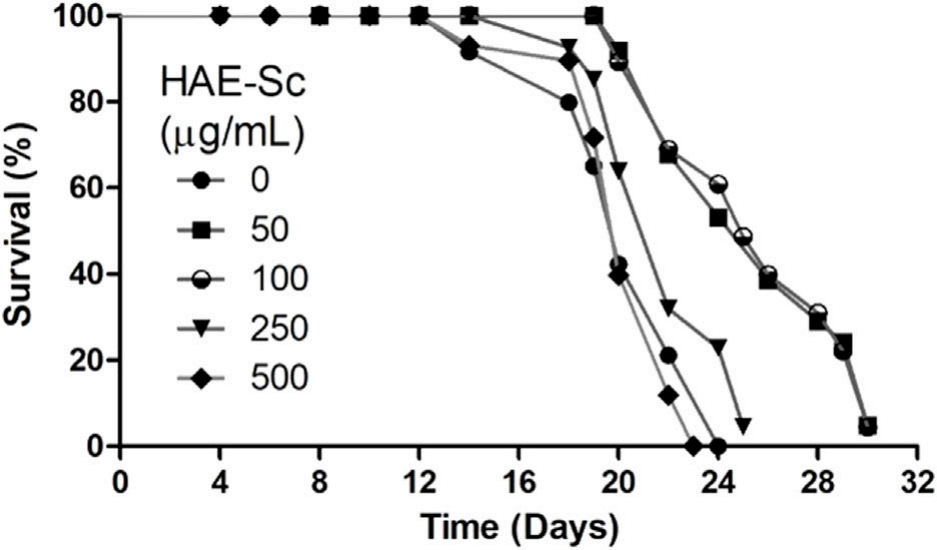
Survival curve of N2 worms grown in presence of HAE-Sc at 20°C.

Longevity curves in N2 *C. elegans* chronically exposed to HAE-Sc at 20°C are displayed in Figure 4. The mean lifespan of the control group was 20.3 days and the maximum 24 days. The maximum lifespan for all treatment groups was 30 days, and this was achieved by worms exposed to 100 μg/mL HAE-Sc, with a mean lifespan of 24 days. The average lifespan was prolonged at 50 and 100 μg/mL HAE-Sc by 15.3% and 18.5%, respectively. Concentrations greater than 500 μg/mL induced death in the worms, and thus, these were not included in the analysis.

### 3.5 Stress resistance assay (heat shock)

**FIGURE 5:**
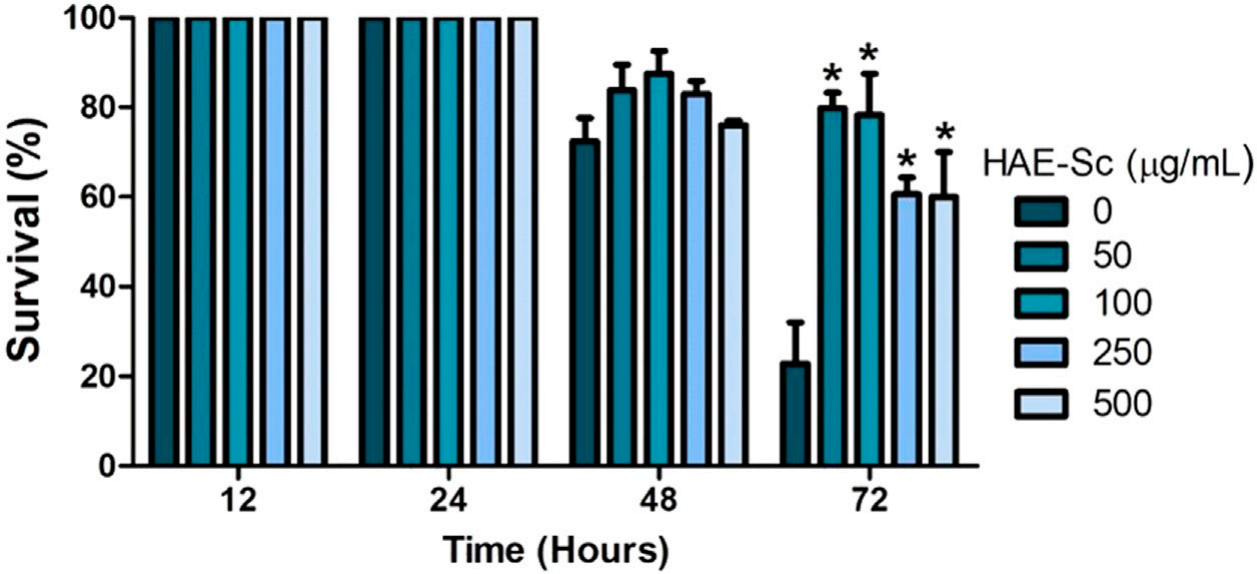
Survival of nematodes at 37°C in the presence of HAE-*Sc*.

The percentage of survival after 2 h of exposure to a temperature of 37°C is depicted in Figure 5, showing that HAE-Sc enhanced the ability of *C. elegans* to resist a high environmental temperature. After 48 h exposure, survival induced by HAE-Sc is not quite evident. However, 72 h after being exposed to 37°C, the survival of the worms exposed to HAE-Sc increased markedly compared to control, especially at lower concentrations (50–100 μg/mL). At 250 and 500 μg/mL, the survival was lower.

### 3.6 Lethality of *C. elegans* exposed to the co-treatment HAE-Sc: Heavy metal (Pb, Hg and Cd)

**FIGURE 6:**
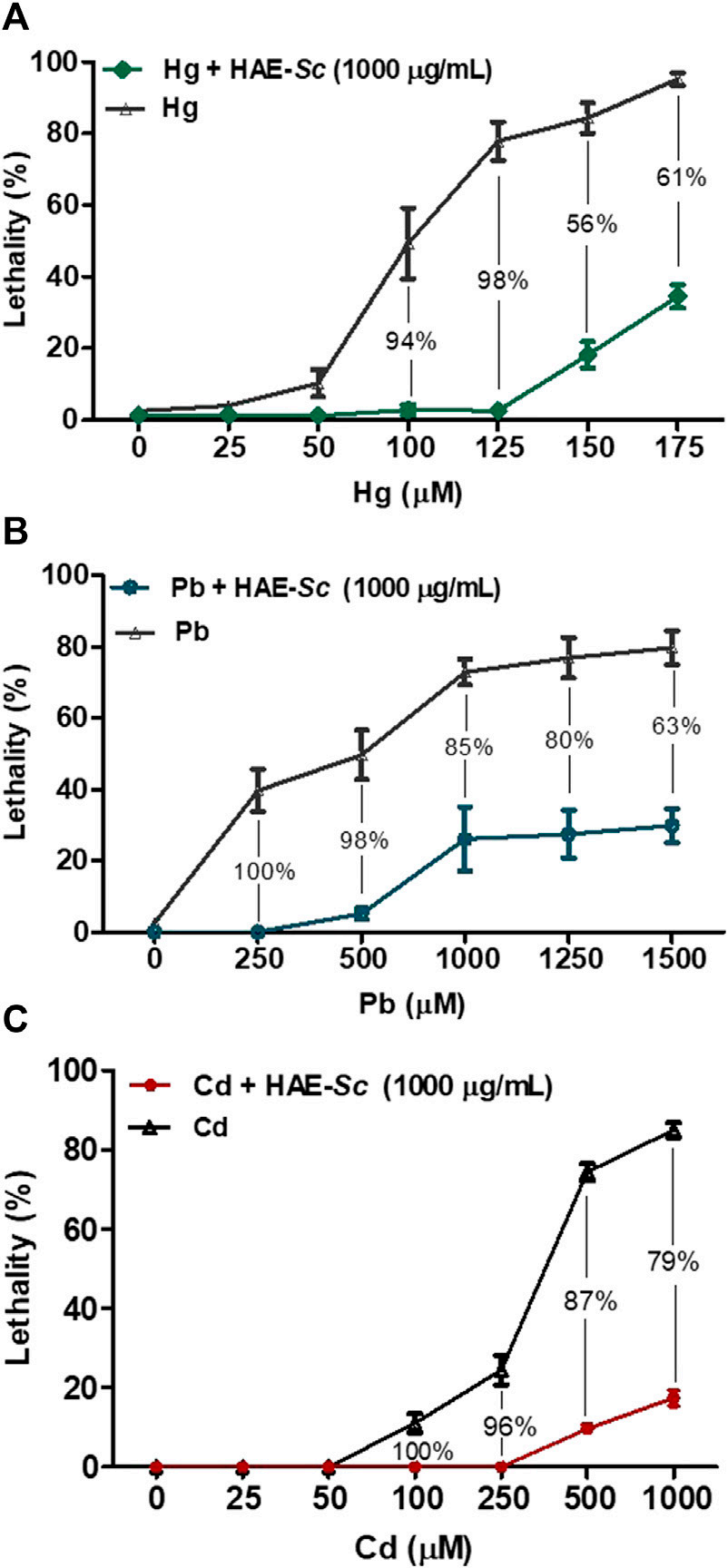
Effect of HAE-Sc on heavy metal-induced lethality in *C. elegans*. **(A–C)** show the lethality in *C. elegans* exposed to Hg, Pb and Cd, respectively. The percentage values represent the reduction in the metal-induced toxicity by the presence of the extract (*p* < 0.05).

The lethality of *C. elegans* exposed to heavy metals after 24 h of treatment are presented in Figure 6. The three heavy metals exhibited high lethality in the nematodes. At the maximum tested concentration, mean lethality rates for Hg (Figure 6A, 175 µM), Pb (Figure 6B, 1500 µM) and Cd (Figure 6C, 1000 µM) were 91.2, 84.9, and 92.4%, respectively. The estimated 24 h-LC_50_ for all three metals, in absence or presence of HAE-Sc (1000 μg/mL) is shown in Table 2.

**TABLE 2:** LC_50_ values for toxic metals alone and in combination with HAE-Sc in *C. elegans*.

LC_50 (95% CI_ ^a^_)_		
Heavy metals	K-Medium	K-medium + HAE-SC
Hg	114 (84–154)	215* (157–293)
Pb	490 (364–660)	2517* (1755–3608)
Cd	265 (177–398)	1450* (737–2853)

^a^ 95% confidence interval. *. Estimated LC_50_.

The co-treatment metal-HAE-Sc (1000 μg/mL) significantly reduced Hg-induced death by 98, 55.9, 60.9% at 125, 150, and 175 μM Hg, respectively (Figure 6A). For Pb-HAE-Sc, lethality was significantly abrogated 98, 85% and 80.1% at 500, 1000 and 1250 μM Pb, respectively. In the case of Cd, the reduction in lethality was 95.9, 87% and 79.5% for 250, 500 and 1000 μM Cd, respectively.

## 4 Discussion

In the present study we used HPLC-ESI-QTOF to characterize the hydroalcoholic extract of *S. cordifolia,* as well as the N2 strain of the nematode *C. elegans* to evaluate its effects on longevity, growth, locomotion, responses to stress caused by high temperatures, and its capacity to reduce heavy metal-induced lethality in the worm. The chemical characterization showed the presence of different chemical groups, all of them with known biological properties. Researchers have corroborated that extracts derived from plants used in traditional medicine contain substances that can improve responses such as life expectancy, reproduction, and the response to oxidative stress in various biological models, including *C. elegans* (Qin et al., 2022).

The most prominent peak corresponded to harman. This alkaloid has a number of pharmacological properties, including acetylcholinesterase and myeloperoxidase inhibition, antianxiety, antidepressant, antioxidant, hypotensive among others, although concomitantly it possesses considerable adverse effects (Khan et al., 2017). Other secondary metabolites include ajmalicine, an alkaloid with potential properties against Alzheimer’s disease (Kashyap et al., 2020); chlorogenic acid has reports for anticancer (Huang et al., 2019), antioxidant and anti-inflammatory activity, hepatic, renal and nervous system protection, and also antibacterial and antitumor properties (Wang et al., 2022); loganine has broad biological activities, such as anti-inflammatory, antioxidant, anti-shock, anti-amnesia and anti-osteoporosis, among others (Xu et al., 2021); 5-caffeoylquinic acids (CQAs) are considered beneficial to human health, mainly for their anti-inflammatory and antioxidant properties (Alcazar-Magaña et al., 2021).

The low lethality of HAE-Sc, together with its low toxicity in terms of reproduction and growth in the *C. elegans* model is noteworthy. *Simira cordifolia* has few reports in databases mostly deal with its geographical distribution and its use as a dye-producing plant (Polesna et al., 2011; Herazo-Vitola et al., 2017).

In addition to the acute toxicity of the extract on the nematode, the study included the assessment of other important toxicological criteria: growth delay, locomotion, and effects on reproduction (Xiong et al., 2017). HAE-*S*c have minor impact on reproduction and growth, HAE-*S*c increases locomotion of *C. elegans,* (Figures 3A–C).

This work showed that HAE-Sc can extend lifespan of *C. elegans*, as also has been observed for a number of plant extracts with anti-oxidative properties, such as those from blueberries (Wang et al., 2018), ginseng (Wang et al., 2021), *Caesalpinia mimosoides* (Rangsinth et al., 2019), and Butiá (*Butia eriospatha*) (Tambara et al., 2020), among other plants. This process has been proposed to be mediated by aging-related pathways that control activation of autophagy, mitochondrial biogenesis and the antioxidant machinery, among other mechanisms (Aman et al., 2021). It is possible that chlorogenic acid, one of the compounds detected in the phytochemical analysis of *S. cordifolia* extract, is linked to the observed results, as its ability to extend the worm’s lifespan and enhance thermotolerance has been previously reported (del valle-Carranza, et al., 2020). Pre-exposure of worms (L1 to L4) to HAE-*S*c ameliorates high temperature-induced lethality, which may be related to the plant species’ ability to enhance the antioxidant system, as observed in other pigmented extracts such as curcumin (Xu et al., 2023). However, more comprehensive research will be necessary to enable the identification of the functional chemical components present in the extract, as well as to understand their interactions.

From a molecular perspective, several studies have demonstrated that daf-16, a transcription factor involved in the regulation of insulin/IGF-1, TOR, AMPK, JNK and germline signals, is a key player in aging and longevity in *C. elegans* (Sun et al., 2017). Moreover, quite recently, it has been suggested that harmol, a beta-carboline molecule reported here, improves mitochondrial function and metabolic parameters, extending healthspan (Costa-Machado et al., 2023). Altogether, the beneficial effects of HAE-Sc on *C. elegans* lifespan may derive from distinct molecular mechanisms linked to the presence of unique pharmacologically active chemicals present in the mixture.

This work tested the effect of HAE-Sc on heavy metal-induced lethality on *C. elegans*. The extract increased the LC_50_ in 1.9-, 5.1- and 5.5-fold for Hg, Pb and Cd, respectively. These elements can alter Fe, Cu, and Zn homeostasis, potentially leading to neurodegenerative diseases, including Alzheimer’s and Parkinson’s disease (Andrade et al., 2017).

The effects of toxic metals on *C. elegans* are well known (Soares et al., 2017). Previous research has demonstrated that the primary mechanism of toxicity of individual heavy metals involves the generation of reactive oxygen species (ROS) through the depletion of glutathione and the binding to sulfhydryl groups of proteins, presenting itself as one of the fundamental cellular responses, even in the nematode *C. elegans* (Hirota and Matsuoka, 2021). In relation to the impact on parameters such as growth and reproduction the relative toxicity of the metals has shown that Hg > Pb > Cd (Huang et al., 2015; Tang et al., 2019), according to our LC_50_ results the acute toxicity of the metals was Hg > Pb > Cd.

The significant effects observed when the lethality induced by the metals was challenged by HAE-Sc is probably due to its antioxidant capacity, also observed for *Haematoxylum brasiletto* (Duran-Izquierdo et al., 2022). As oxidative stress participates in the pathogenesis of many diseases in humans (Kim et al., 2021) the extract offers an interesting opportunity for therapeutic uses.

The chemical mixture in HAE-Sc may not only interfere with the oxidative stress induced by the metals, it also provides metabolites, such as harman, with protective actions on neurological function via anti-inflammatory effects (Piechowska et al., 2019) or by inhibition of monoamine oxidase, working as antidepressant (Herraiz, 2023). This action of HAE-Sc may be extrapolated to the control of neurodegenerative diseases, where beneficial impacts on oxidative stress, inflammation and neuronal stimulation are main objectives of treatments (Murphy and Hartley, 2018; Peng et al., 2022).

In summary, we first reported the phytochemical composition of *S. cordifolia.* The results from the *in vivo* assays evidenced the potential of compounds from HAE-Sc, to neutralize the damage in *C. elegans* produced by heavy metals or heat, the extract also prolongs the half-life of the worms under normal conditions. Further studies are needed to elucidate the signaling pathways involved in the oxidative stress resistance of *C. elegans* treated with *S. cordifolia*. Likewise, it will be necessary to use transgenic strains that allow the identification of molecular mechanisms involved in the nematode’s response to *S. cordifolia*.

The main limitations of the study lie in the need to elucidate the underlying mechanisms in the nematode’s response to the extract and its beneficial effects, likely through transcriptomic analyses of the worm. Additionally, it is necessary to conduct research related to the evaluation of other plant parts extracts using fractions with different polarities, in order to identify compounds with higher biological activity.

## 5 Conclusion

The present study evaluated the physiological effects of HAE-Sc using the *C. elegans* biological model. The findings revealed that the extract exerts minimal but significant toxic effects on physiological parameters of the worm in a dose-dependent manner, even though it has a low lethality. Interestingly, it provokes an increase in locomotion in the nematode, suggesting neurological activation. The HAE-Sc prolongs the lifespan, protects against temperature-induced stress, and pre-exposure of the worms to the extract diminishes lethality induced by toxic heavy metals.

## Data Availability

The original contributions presented in the study are included in the article/Supplementary Material, further inquiries can be directed to the corresponding author.
