# Supplementary material for: Simira cordifolia protects against metal induced-toxicity in Caenorhabditis elegans
Source: Front Pharmacol. 2023 Nov 15;14:1235190. doi: 10.3389/fphar.2023.1235190 (PMC10684763; doi:10.3389/fphar.2023.1235190)
Supplement: Supplementary file 1 [file DataSheet1.docx]

Supplementary Material

*Simira cordifolia* protects against metal induced-toxicity in *Caenorhabditis elegans*

Margareth Duran-Izquierdo^1^, Lucellys Sierra-Marquez^1^, Maria Taboada-Alquerque^1^, and Jesus Olivero-Verbel^1^

1. Environmental and Computational Chemistry Group, School of Pharmaceutical Sciences, Zaragocilla Campus, University of Cartagena, Cartagena, 130014, Colombia.

Correspondence:

Prof. Jesus Olivero-Verbel. Ph.D.

Environmental and Computational Chemistry Group

School of Pharmaceutical Sciences

Zaragocilla Campus

University of Cartagena

Cartagena, 130015.

Colombia

E-mail: [joliverov@unicartagena.edu.co](mailto:joliverov@unicartagena.edu.co)

Phone: 57-3185359815

# Supplementary Figures and Tables

Table S1. Fragment ions of chemical constituents tentatively identified in the *S. cordifolia* by HPLC-QTOF-

MS/MS.

| No. Fig. 1 | Positive ion fragments | | | Identification  criteria | References |
| --- | --- | --- | --- | --- | --- |
|  | **Fragment type** | **Fragment**  **formula** | **m/z** |  |  |
| 1 | [M+Na]^+^  [M-C_6_H_11_O_4_Na]^+^ | [C_19_H_28_O_13_Na]^+^  [C_13_H_17_O_9_]^+^ | 487.1411  317.0799 | a | (Martins and Nunez, 2015) |
| 2 | [M+Na]^+^  [M-C_7_H_16_O_5_Na]^+^ | [C_17_H_26_O_10_Na]^+^  [C_10_H_10_O_5_]^+^ | 413.1421  210.0513 | b | (Berger et al., 2015; Mass Bank code: TY000062) |
| 3 | [M+H]^+^  [M+H-C_2_H_4_O]^+^ | [C_12_H_18_NO_3_]^+^  [C_10_H_14_NO_2_]^+^ | 224.1279  180.1012 |  |  |
| 4 | [M+H]^+^  [M+H-C_7_H_12_O_6_]^+^  [M+H-C_8_H_12_O_7_]^+^ | [C_16_H_19_O_9_]^+^  [C_9_H_7_O_3_]^+^  [C_8_H_7_O_2_]^+^ | 355.1021  163.0383  135.0443 | b | Mass Bank code: FIO00622 |
| 5 | [M+H]^+^  [M+H-CO]^+^  [M+H-C_3_H_5_N_2_]^+^ | [C_12_H_11_N_2_O]^+^  [C_11_H_11_N_2_]^+^  [C_9_H_6_O]^+^ | 199.0858  171.0912  131.0483 |  |  |
| 6 | [M+H]^+^  [M+H-C_6_H_10_O_5_]^+^  [M+H-C_7_H_10_O_7_]^+^ | [C_26_H_29_N_2_O_9_]^+^  [C_20_H_19_N_2_O_4_]^+^  [C_19_H_19_N_2_O_2_]^+^ | 513.1877  351.1335  307.1444 | a | (Martins and Nunez, 2015) |
| 7 | [M+H]^+^  [M+H-C_7_H_12_O_6_]^+^ | [C_16_H_19_O_9_]^+^  [C_9_H_7_O_3_]^+^ | 355.1303  163.0384 | b | (Veeren et al., 2020; Mass Mass Bank code: FIO00622) |
| 8 | [M+H]^+^  [M+H-C_7_H_12_O_6_]^+^  [M+H-C_8_H_16_O_7_]^+^ | [C_17_H_21_O_9_]^+^  [C_10_H_9_O_3_]^+^  [C_9_H_5_O_2_]^+^ | 369.1471  177.0543  145.0280 | b | (Veeren et al., 2020; Mass Bank code: CCMSLIB00000853669) |
| 9 | [M+H]^+^  [M+H-C_6_H_10_O_5_]^+^  [M+H-C_6_H_12_O_6_]^+^  [M+H-C_10_H_16_O_6_]^+^ | [C_16_H_23_O_9_]^+^  [C_10_H_13_O_4_]^+^  [C_10_H_11_O_3_]^+^  [C_6_H_7_O_3_]^+^ | 359.1421  197.0801  179.0700  127.0382 | b | (Martins and Nunez, 2015; Mass Bank code: CCMSLIB00000852242) |
| 10 | [M+H]^+^  [M+H-C_6_H_10_O_5_]^+^  [M+H-C_6_H_12_O_6_]^+^  [M+H-C_10_H_18_O_8_]^+^ | [C_16_H_27_O_8_]^+^  [C_10_H_17_O_3_]^+^  [C_10_H_15_O_2_]^+^  [C_6_H_9_]^+^ | 347.1696  185.1172  167.1049  81.0701 | a | (Xie et al., 1988) |
| 11 | [M+H]^+^  [M+H-C_8_H_16_O_8_]^+^  [M+H-C_17_H_24_O_10_]^+^  [M+H-C_18_H_28_O_11_]^+^ | [C_27_H_33_O_13_]^+^  [C_19_H_17_O_5_]^+^  [C_10_H_9_O_3_]^+^  [C_9_H_5_O_2_]^+^ | 565.1918  325.1026  177.0539  145.0282 | a | (Chen et al., 2016) |
| 12 | [M+H]^+^  [M+H-H_2_O]^+^  [M+H-C_7_H_9_O_2_]^+^ | [C_19_H_21_N_2_O_3_]^+^  [C_19_H_19_N_2_O_2_]^+^  [C_12_H_10_N_2_]^+^ | 325.1551  307.1446  182.0837 |  |  |
| 13 | [M+H]^+^  [M+H-CH_3_]^+^  [M+H-C_3_H_4_N_2_]^+^ | [C_12_H_11_N_2_]^+^  [C_11_H_8_N_2_]^+^  [C_9_H_7_]^+^ | 183.0917  168.0681  115.0542 | b | (Martins and Nunez, 2015; Mass Bank code: FIO00111) |
| 14 | [M+H]^+^  [M+H-C_8_H_15_NO_5_]^+^  [M+H-C_17_H_25_NO_9_]^+^ | [C_27_H_35_N_2_O_9_]^+^  [C_19_H_20_NO_4_]^+^  [C_10_H_10_N]^+^ | 531.2331  326.1387  144.0812 |  |  |
| 15 | [M+H]^+^  [M+H-H_2_O]^+^  [M+H-C_7_H_9_O_4_]^+^ | [C_19_H_19_N_2_O_4_]^+^  [C_19_H_17_N_2_O_3_]^+^  [C_12_H_10_N_2_]^+^ | 339.1345  321.1243  182.0827 |  |  |
| 16 | [M+H]^+^  [M+H-C_2_H_6_O]^+^  [M+H-C_3_H_8_O_3_]^+^  [M+H-C_9_H_15_O_3_]^+^ | [C_21_H_25_N_2_O_3_]^+^  [C_19_H_19_N_2_O_2_]^+^  [C_18_H_17_N_2_]^+^  [C_12_H_10_N_2_]^+^ | 353.1867  307.1447  261.1380  182.0839 |  |  |
| 17 | [M+H]^+^  [M+H-C_2_H_5_N]^+^  [M+H-C_9_H_15_NO]^+^ | [C_19_H_25_N_2_O]^+^  [C_17_H_20_NO]^+^  [C_10_H_10_N]^+^ | 297.1958  254.1541  144.0800 | a | (Martins and Nunez, 2015) |
| 18 | [M+H]^+^  [M+H-C_9_H_8_NO_2_]^+^  [[M+H-C_15_H_20_O_8_]^+^ | [C_26_H_31_N_2_O_8_]^+^  [C_17_H_23_NO_6_]^+^  [C_11_H_11_N_2_]^+^ | 499.1859  337.1530  171.0911 | b | Mass Bank code: CCMSLIB00004680054 |

Collision energy used to these fragments was 20 eV

^a^ Tentative identification based on m/z positive mode, reported in the literature for *Simira genus* or other species of the Rubiaceae family (Ram, et al., 2004; Vinicius et al., 2014; Martins & Nunez, 2015)

^b^ Tentative identification based on the fragmentation pattern study (ESI-QTOF) and data reported in scientific articles.

## Supplementary Figures

Figure S1. Compound fragment spectrum results (MS/MS)

**Positive ionization mode: Compound fragment spectrum results (MS/MS)**

| **Compound 1**  Retention time: 8.754  Monoisotopic mass: 464.1520  Formula: C_19_H_28_O_13_  Tentative annotation: Diderroside  Compound class: Iridoid | 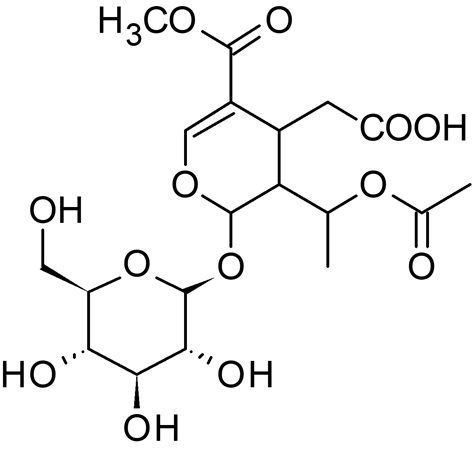 |
| --- | --- |

| **Compound 2**  Retention time: 8.931  Monoisotopic mass: 390.1516  Formula: C_17_H_26_O_10_  Tentative annotation: Loganin  Compound class: Iridoid | 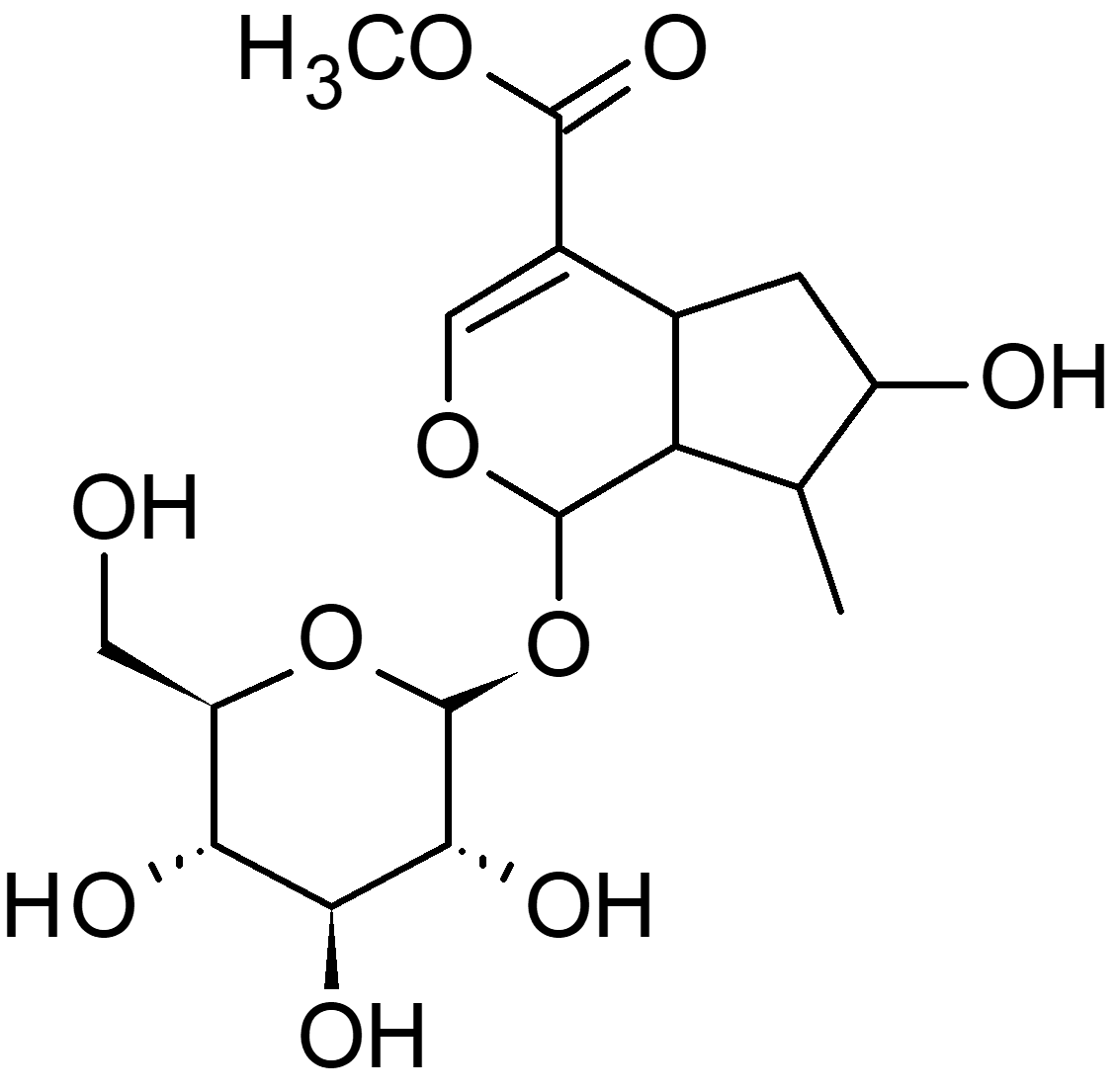 |
| --- | --- |

| **Compound 3**  Retention time: 9.054  Monoisotopic mass: 223.1208  Formula: C_12_H_17_NO_3_  Tentative annotation: Anhalonidine  Compound class: Isoquinoline Alkaloid | 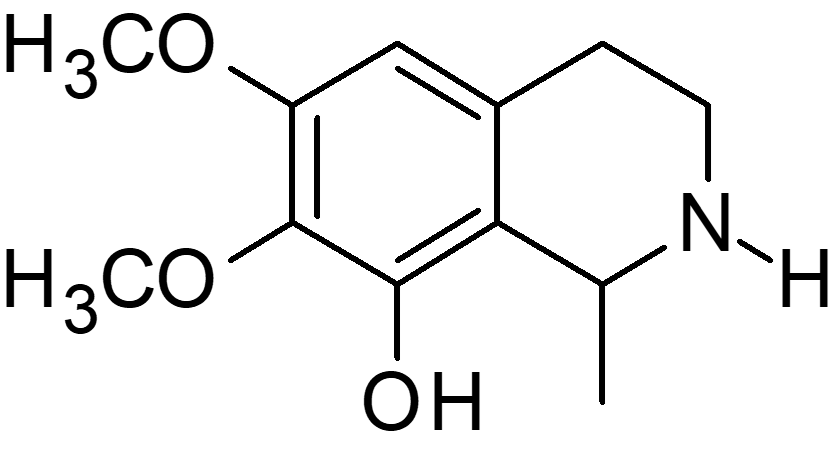 |
| --- | --- |

| **Compound 4**  Retention time: 9.41  Monoisotopic mass: 354.0949  Formula: C_16_H_18_O_9_  Tentative annotation: Chlorogenic acid  Compound class: Polyphenol | 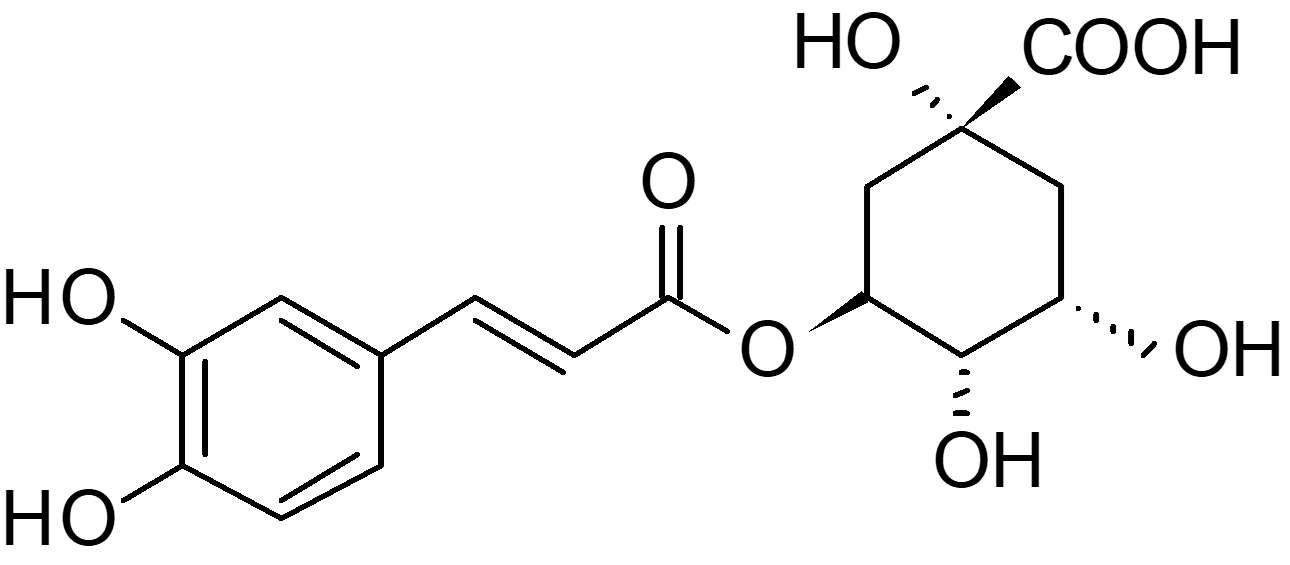 |
| --- | --- |

| **Compound 5**  Retention time: 9.602  Monoisotopic mass: 198.0790  Formula: C_12_H_10_N_2_O  Tentative annotation: Harmol  Compound class: 𝛽-carboline alkaloid | 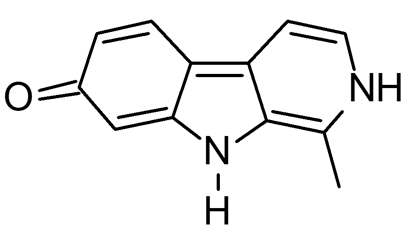 |
| --- | --- |


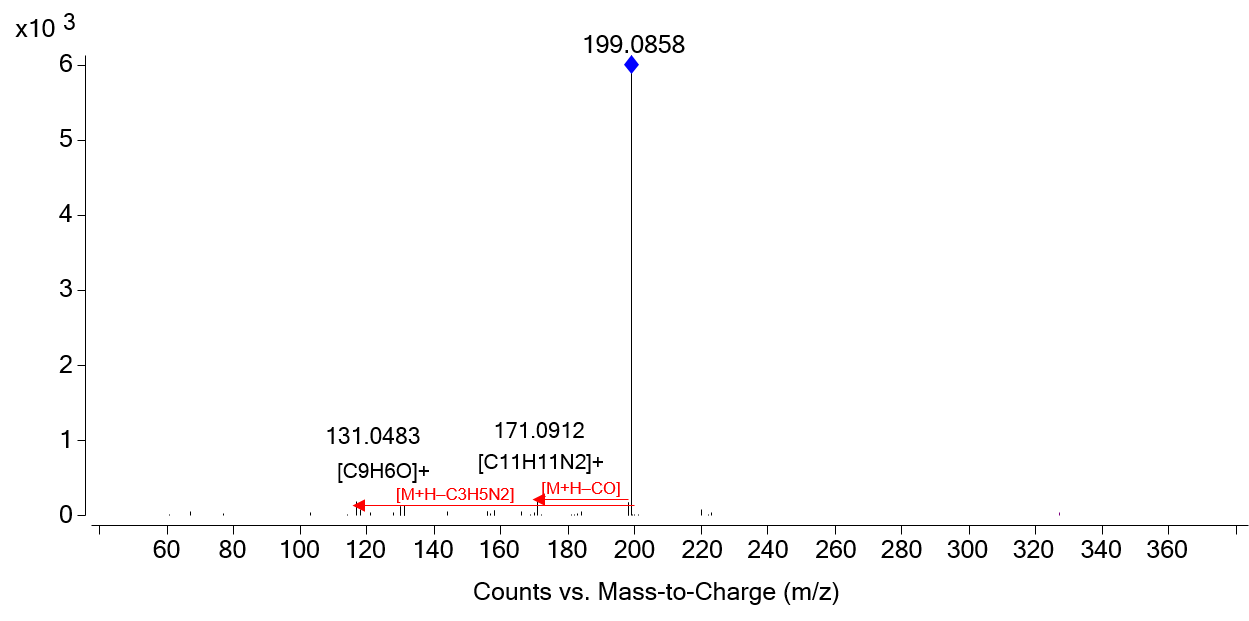


| **Compound 6**  Retention time: 9.671  Monoisotopic mass: 512.1808  Formula: C_26_H_28_N_2_O_9_  Tentative annotation: Lyalosidic acid  Compound class: 𝛽-carboline glucoalkaloid | 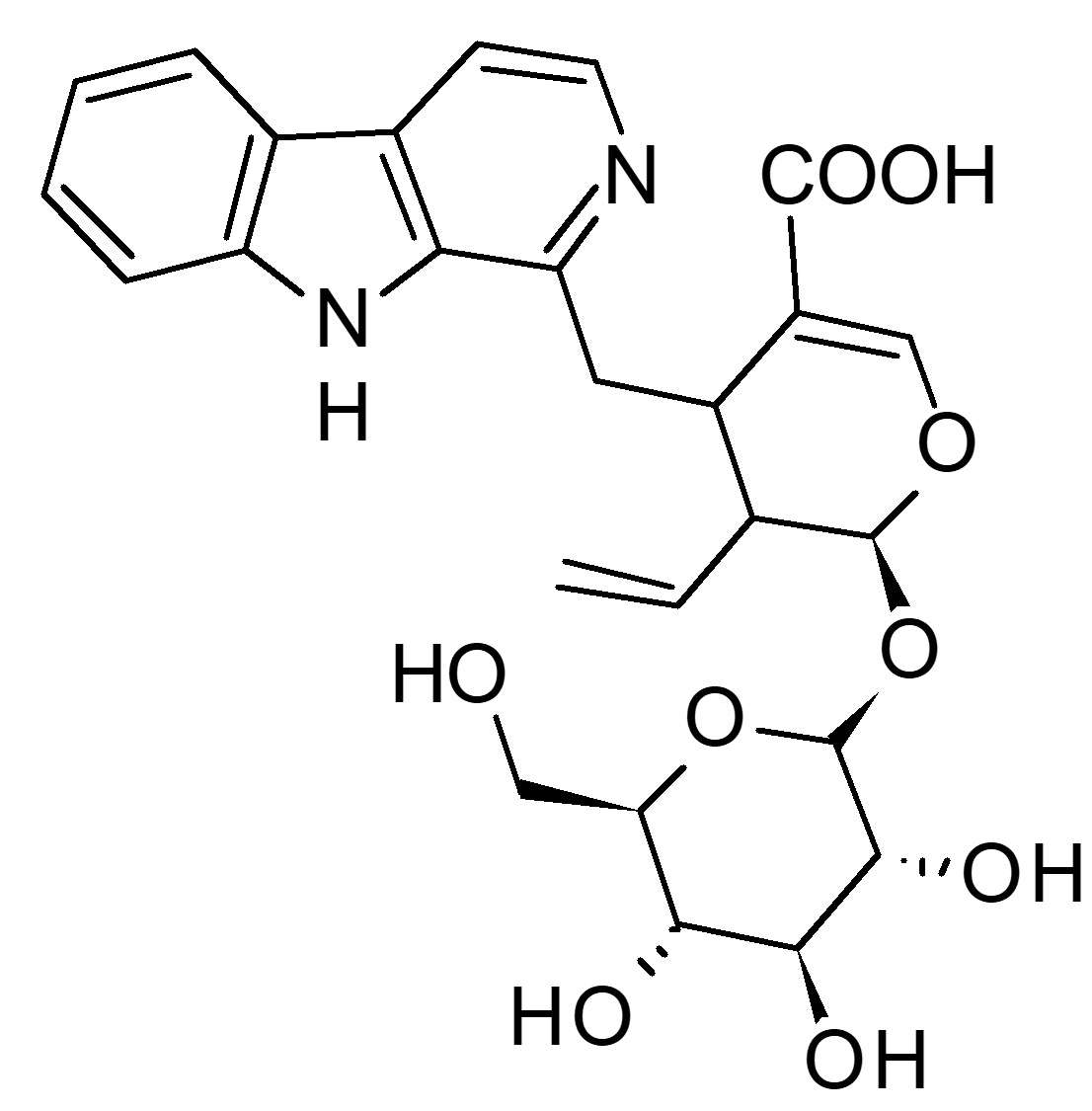 |
| --- | --- |


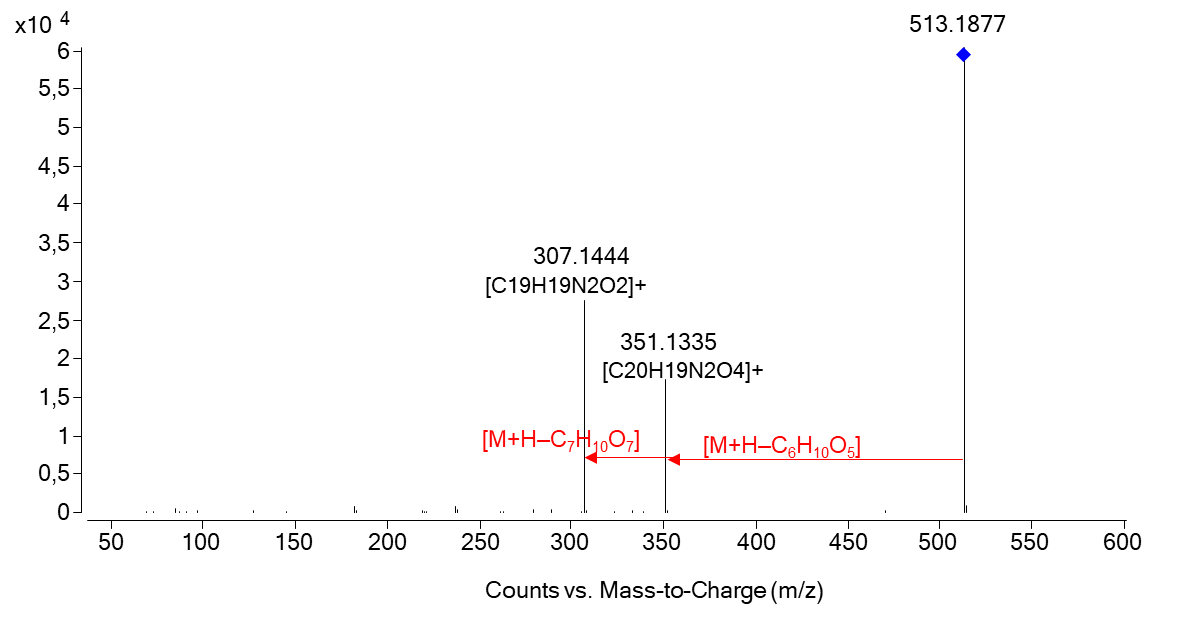


| **Compound 7**  Retention time: 9.795  Monoisotopic mass: 354.0949  Formula: C_16_H_18_O_9_  Tentative annotation: Chlorogenic acid isomer  Compound class: Polyphenol | 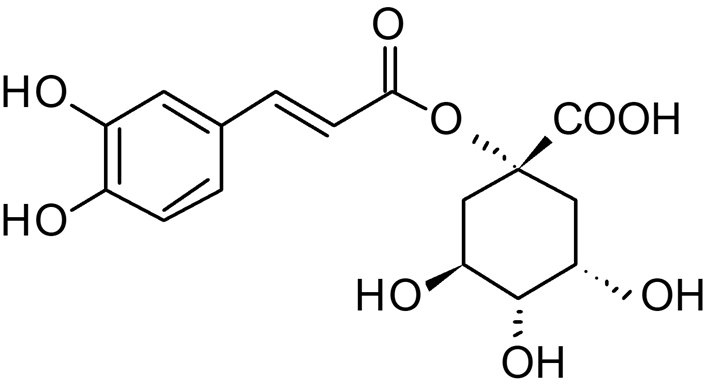 |
| --- | --- |

| **Compound 8**  Retention time: 10.01  Monoisotopic mass: 368.1117  Formula: C_17_H_20_O_9_  Tentative annotation: Feruloylquinic acid  Compound class: Polyphenol | 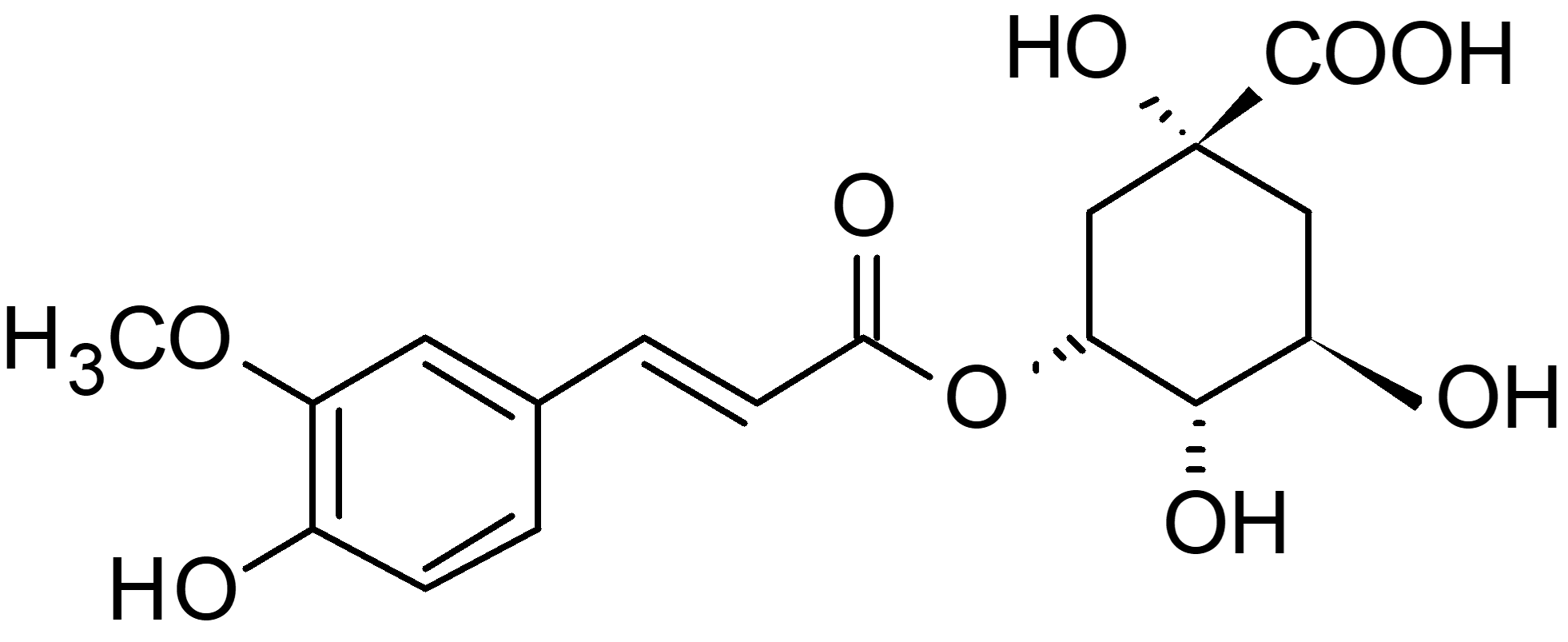 |
| --- | --- |

| **Compound 9**  Retention time: 10.027  Monoisotopic mass: 358.1269  Formula: C_16_H_22_O_9_  Tentative annotation: Sweroside  Compound class: Iridoid | 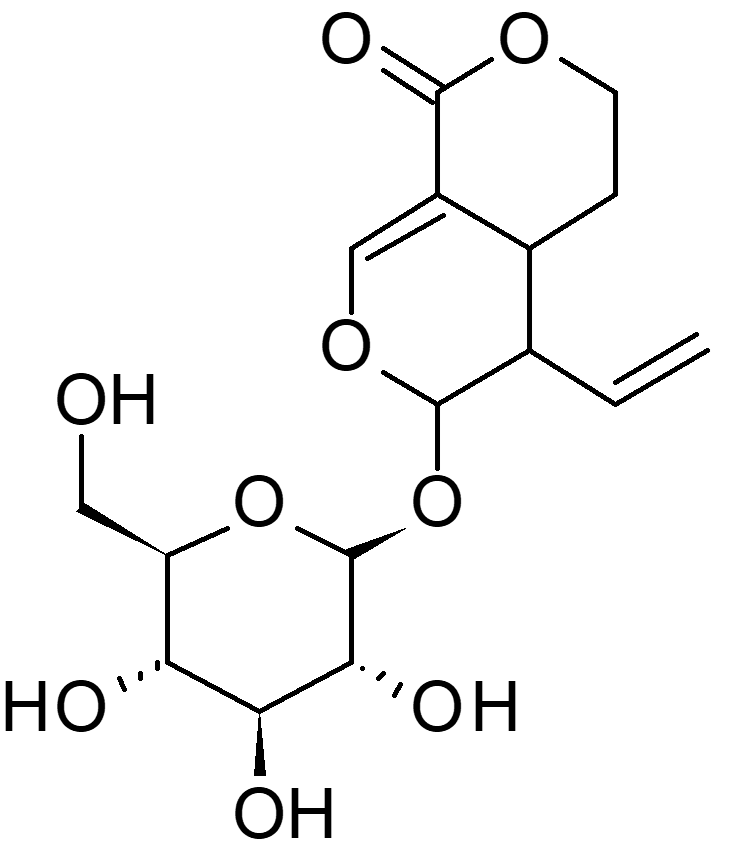 |
| --- | --- |


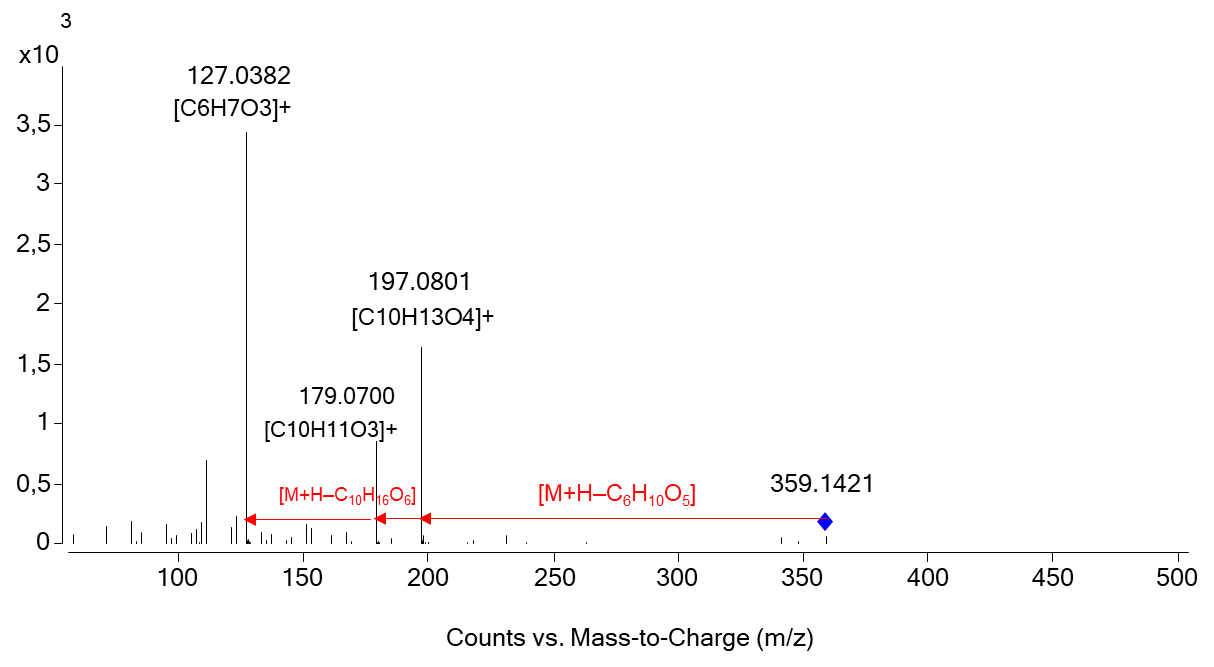


| **Compound 10**  Retention time: 10.281  Monoisotopic mass: 346.1623  Formula: C_16_H_26_O_8_  Tentative annotation: Nepetaside  Compound class: Iridoid | 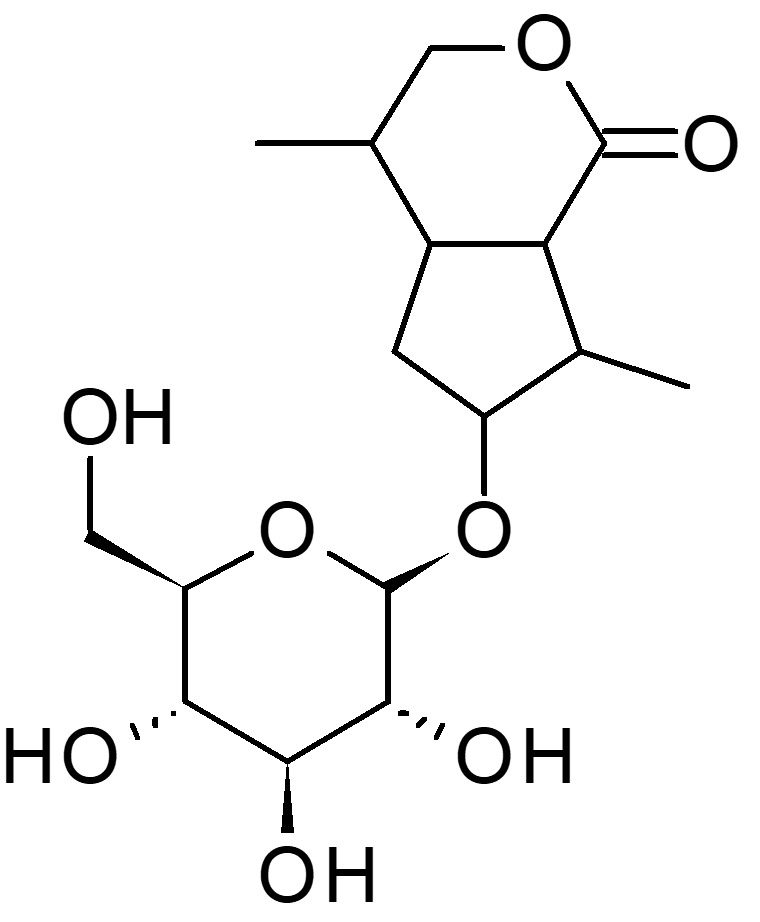 |
| --- | --- |

| **Compound 11**  Retention time: 10.281  Monoisotopic mass: 564.1864  Formula: C_27_H_32_O_13_  Tentative annotation: 6-O-methoxylcinnamoyl scandoside  Compound class: Iridoid ester | 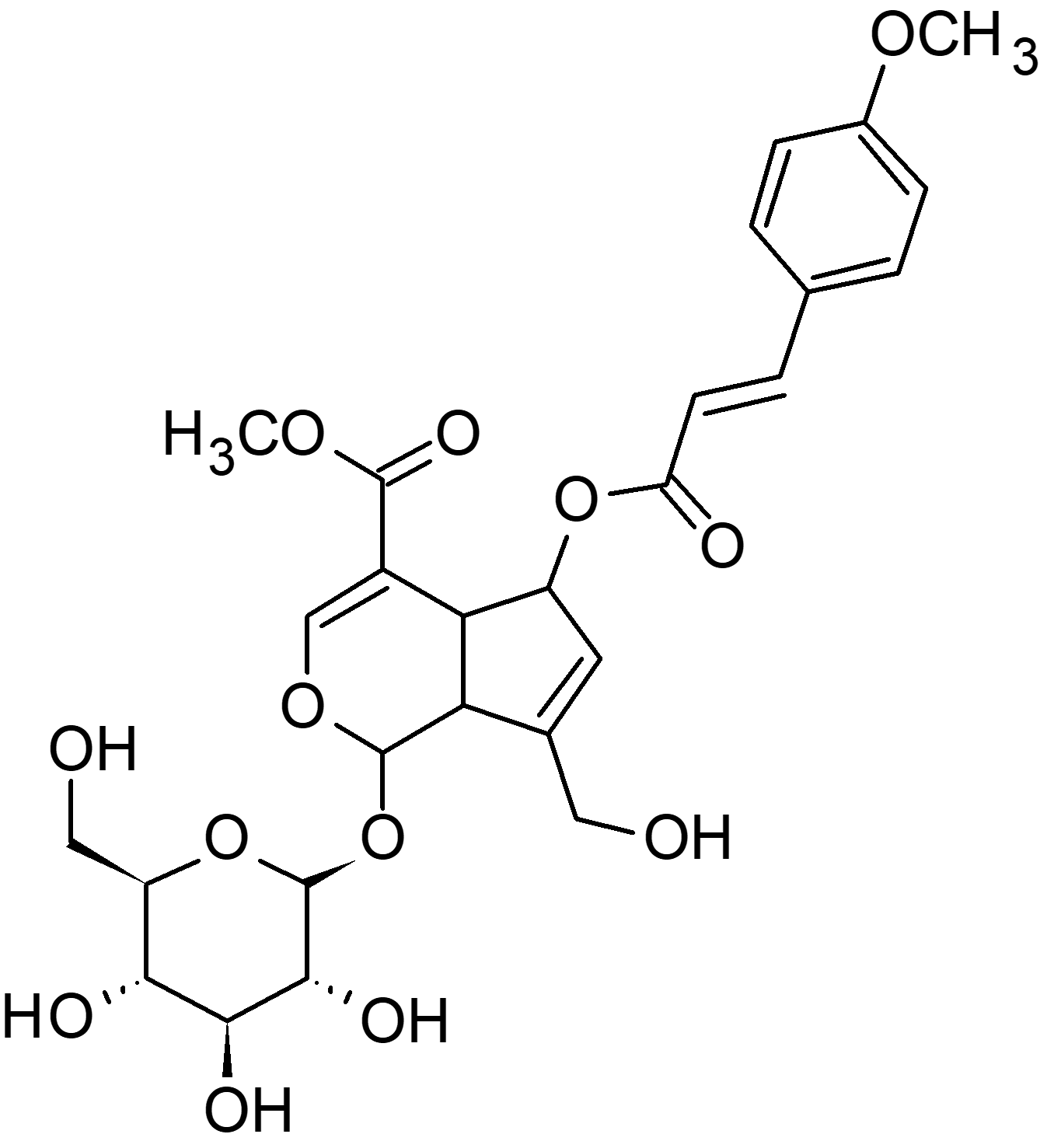 |
| --- | --- |

| **Compound 12**  Retention time: 10.35  Monoisotopic mass: 324.1837  Formula: C_19_H_20_N_2_O_3_  Tentative annotation: 𝛽-carboline alkaloid type  Compound class: 𝛽-carboline alkaloid | |  | | |
| --- | --- | --- | --- | --- |
| **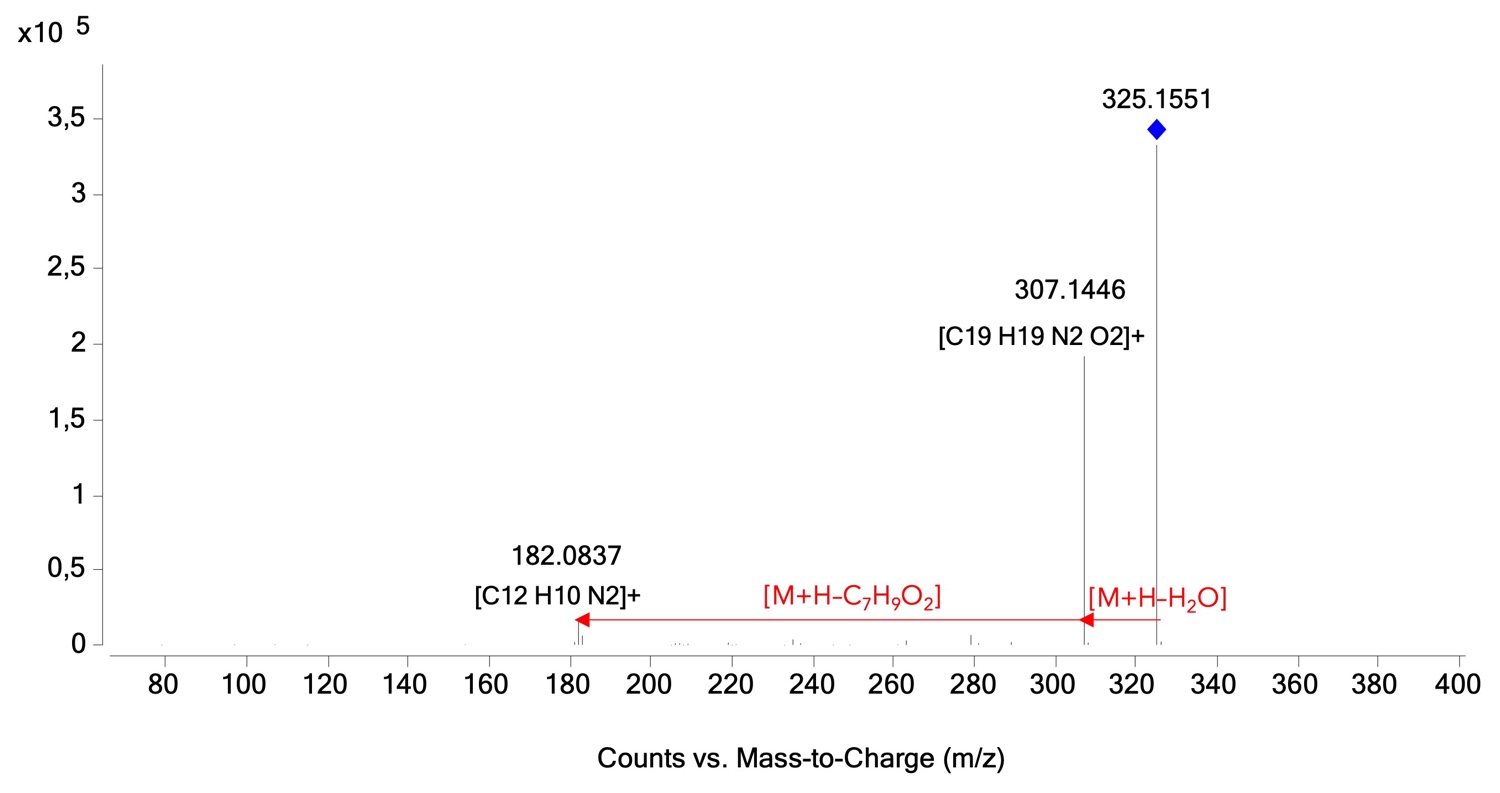** | | | |  |
| **Compound 13**  Retention time: 10.431  Monoisotopic mass: 182.0847  Formula: C_12_H_10_N_2_  Tentative annotation: Harmane  Compound class: 𝛽-carboline alkaloid | 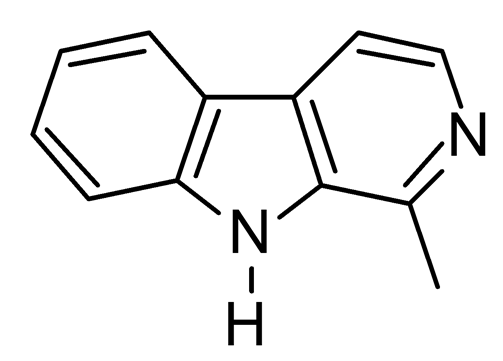 | |  |  |

| **Compound 14**  Retention time: 10.51  Monoisotopic mass: 530.2267  Formula: C_27_H_34_N_2_O_9_  Tentative annotation: indole glucoalkaloid type  Compound class: indole glucoalkaloid |  |
| --- | --- |

| **Compound 15**  Retention time: 10.712  Monoisotopic mass: 338.1271  Formula: C_19_H_18_N_2_O_4_  Tentative annotation: Alamarine  Compound class: isoquinolinonaphthyridine alkaloid | 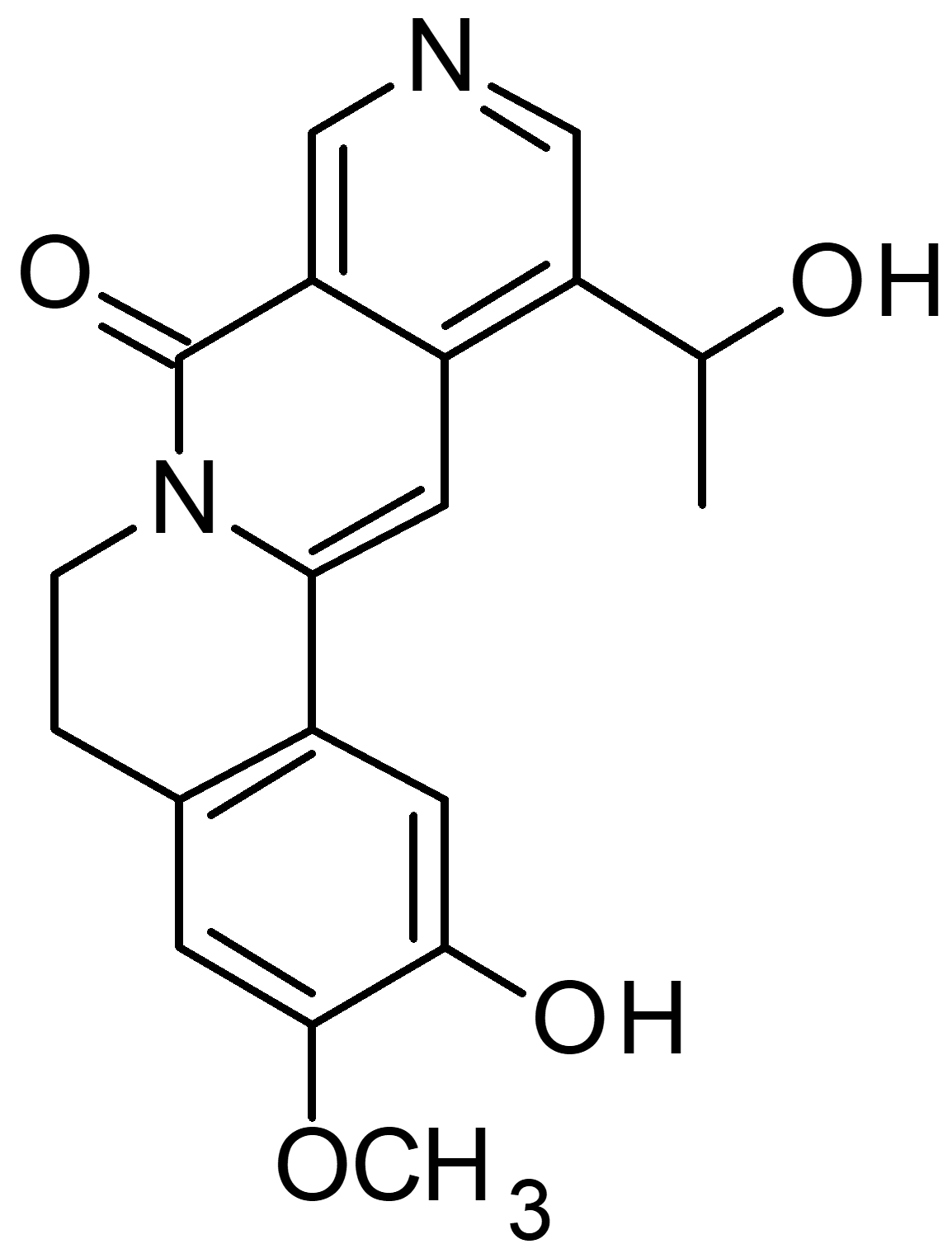 |
| --- | --- |

| **Compound 16**  Retention time: 10.971  Monoisotopic mass: 352.1795  Formula: C_21_H_24_N_2_O_3_  Tentative annotation: 19-Epi-Ajmalicine  Compound class: Indole alkaloid | 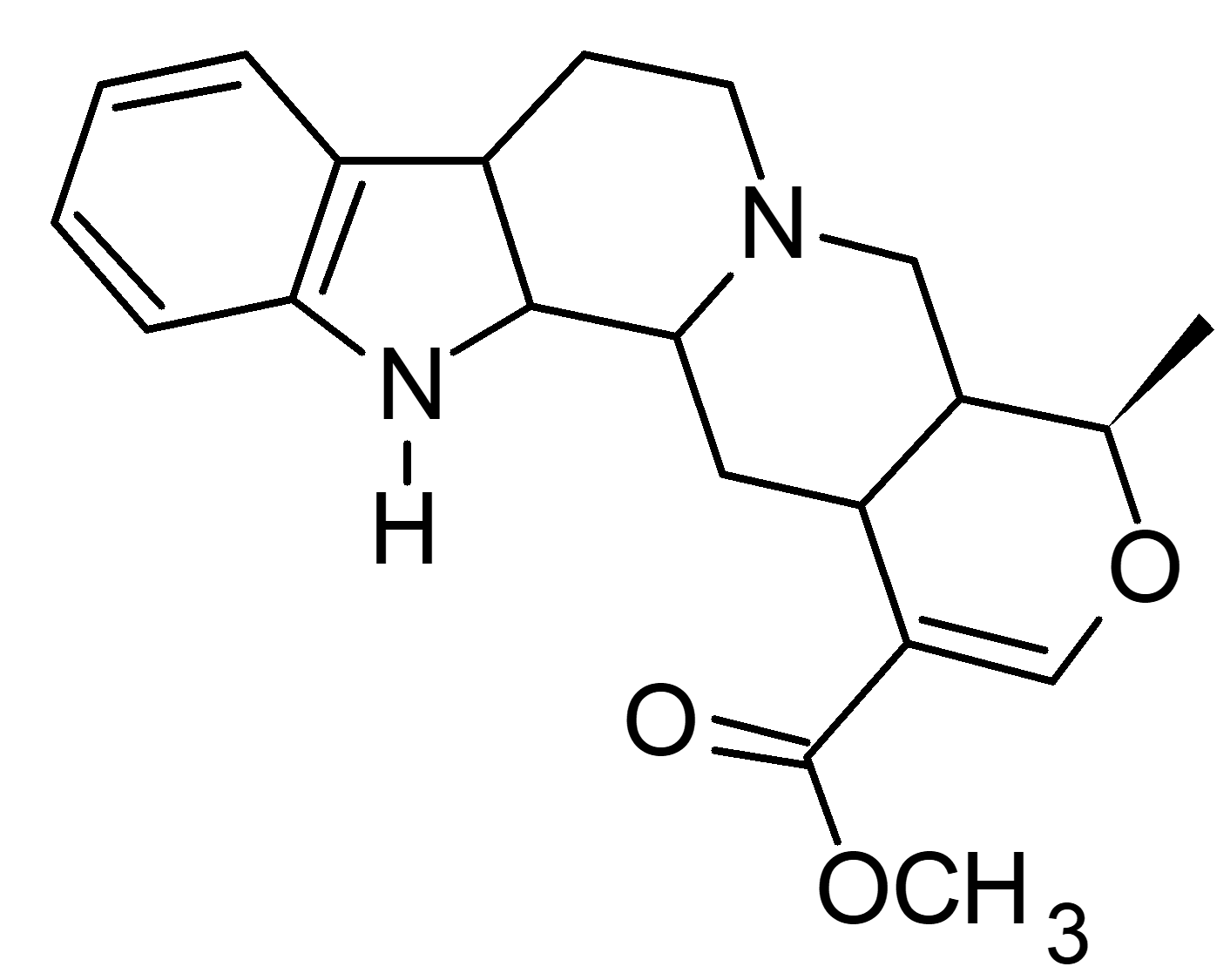 |
| --- | --- |

| **Compound 17**  Retention time: 11.552  Monoisotopic mass: 296.1888  Formula: C_19_H_24_N_2_O  Tentative annotation: No ID  Compound class: Indole alkaloid |  |
| --- | --- |

| **Compound 18**  Retention time: 11.840  Monoisotopic mass: 498.2019  Formula: C_26_H_30_N_2_O_8_  Tentative annotation: Strictosamide  Compound class: Indole glucoalkaloid | 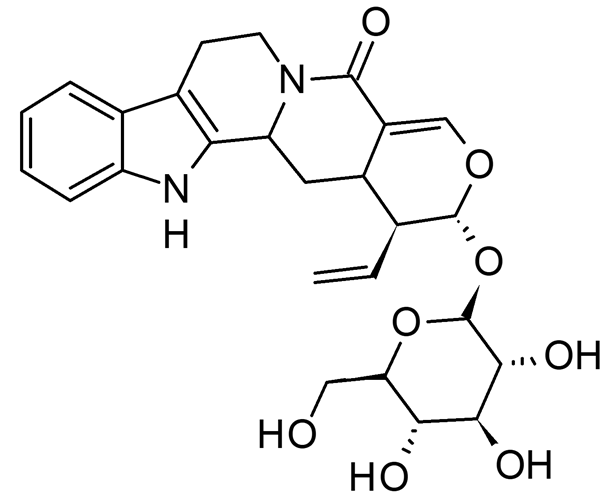 |
| --- | --- |

**References**

Berger, A., Kostyan, M. K., Klose, S. I., Gastegger, M., Lorbeer, E., Brecker, L., et al. (2015). Loganin and secologanin derived tryptamine–iridoid alkaloids from Palicourea crocea and Palicourea padifolia (Rubiaceae). *Phytochem*. 116, 162-169. doi: 10.1016/j.phytochem.2015.05.013

Chen, R., He, J., Tong, X., Tang, L., and Liu, M. (2016). The Hedyotis diffusa Willd.(Rubiaceae): a review on phytochemistry, pharmacology, quality control and pharmacokinetics. *Mol.* 21(6), 710. doi: 10.3390/molecules21060710

Martins, D., and Nunez, C. V. (2015). Secondary metabolites from Rubiaceae species. *Mol*. 20(7), 13422-13495. doi: 10.3390/molecules200713422

Phuong, N. M., Sung, T. V., Schmidt, J., and, A. P., and Adam, G. (1998). Capitelline-A new indole alkaloid from Hedyotis capitellata. *Nat. prod. Lett.* 11(2), 93-100. doi: 10.1177/1934578X211047705

Veeren, B., Ghaddar, B., Bringart, M., Khazaal, S., Gonthier, M. P., Meilhac, O., et al. (2020). Phenolic profile of herbal infusion and polyphenol-rich extract from leaves of the medicinal plant antirhea borbonica: toxicity assay determination in zebrafish embryos and larvae. *Mol*. 25(19), 4482. doi: 10.3390/molecules25194482

Xie, S., Uesato, S., Inouye, H., Fujita, T., Murai, F., Tagawa, M., et al. (1988). Absolute structure of nepetaside, a new iridoid glucoside from Nepeta cataria. *Phytochem.* 27(2), 469-472. doi: 10.1016/0031-9422(88)83122-4
